# Free Levels of Selected Organic Solutes and Cardiovascular Morbidity and Mortality in Hemodialysis Patients: Results from the Retained Organic Solutes and Clinical Outcomes (ROSCO) Investigators

**DOI:** 10.1371/journal.pone.0126048

**Published:** 2015-05-04

**Authors:** Tariq Shafi, Timothy W. Meyer, Thomas H. Hostetter, Michal L. Melamed, Rulan S. Parekh, Seungyoung Hwang, Tanushree Banerjee, Josef Coresh, Neil R. Powe

**Affiliations:** 1 Department of Medicine, Division of Nephrology, Johns Hopkins University, Baltimore, Maryland, United States of America; 2 Welch Center for Prevention, Epidemiology and Clinical Research, Johns Hopkins University, Baltimore, Maryland, United States of America; 3 Department of Medicine, Division of Nephrology, Veterans Administration Palo Alto Health Care System and Stanford University, Palo Alto, California, United States of America; 4 Department of Medicine, Case Western University School of Medicine, Cleveland, Ohio, United States of America; 5 Departments of Medicine and Epidemiology & Population Health, Albert Einstein College of Medicine, Bronx, New York, United States of America; 6 Departments of Medicine and Pediatrics, University of Toronto, Toronto, Canada; 7 Department of Medicine, University of California San Francisco, San Francisco, California, United States of America; 8 Departments of Epidemiology and Biostatistics, Johns Hopkins Bloomberg School of Public Health, Baltimore, Maryland, United States of America; Medical University of Graz, AUSTRIA

## Abstract

**Background and Objectives:**

Numerous substances accumulate in the body in uremia but those contributing to cardiovascular morbidity and mortality in dialysis patients are still undefined. We examined the association of baseline free levels of four organic solutes that are secreted in the native kidney — p-cresol sulfate, indoxyl sulfate, hippurate and phenylacetylglutamine — with outcomes in hemodialysis patients.

**Design, Setting, Participants and Measurements:**

We measured these solutes in stored specimens from 394 participants of a US national prospective cohort study of incident dialysis patients. We examined the relation of each solute and a combined solute index to cardiovascular mortality and morbidity (first cardiovascular event) using Cox proportional hazards regression adjusted for demographics, comorbidities, clinical factors and laboratory tests including Kt/V_UREA_.

**Results:**

Mean age of the patients was 57 years, 65% were white and 55% were male. In fully adjusted models, a higher p-cresol sulfate level was associated with a greater risk (HR per SD increase; 95% CI) of cardiovascular mortality (1.62; 1.17–2.25; p=0.004) and first cardiovascular event (1.60; 1.23–2.08; p<0.001). A higher phenylacetylglutamine level was associated with a greater risk of first cardiovascular event (1.37; 1.18–1.58; p<0.001). Patients in the highest quintile of the combined solute index had a 96% greater risk of cardiovascular mortality (1.96; 1.05–3.68; p=0.04) and 62% greater risk of first cardiovascular event (1.62; 1.12–2.35; p=0.01) compared with patients in the lowest quintile. Results were robust in sensitivity analyses.

**Conclusions:**

Free levels of uremic solutes that are secreted by the native kidney are associated with a higher risk of cardiovascular morbidity and mortality in incident hemodialysis patients.

## Introduction

Cardiovascular disease is the leading cause of morbidity and mortality in dialysis patients but accumulated substances that contribute to cardiovascular morbidity and mortality remain undefined. [1–5] These substances are often referred to as organic uremic solutes or uremic toxins. They vary greatly in their size, distribution and protein binding.[3, 6] Urea, the current standard for dialysis adequacy, is a non-toxic uremic solute that is easily removed by dialysis. Conventional dialysis maintains plasma urea levels within five to ten times normal. Levels of many non-urea solutes, however, are much less effectively controlled by Kt/V_UREA_-guided intermittent dialysis schedules.[4, 6–8] Such solutes may contribute to the persistent uremic symptoms and accelerated cardiovascular disease that has been referred to as the “Residual Uremic Syndrome”.[9]

The current study examined four solutes—p-cresol sulfate, indoxyl sulfate, hippurate and phenylacetylglutamine—that share the property of colon microbial derivation,[10–14] rapid clearance by secretion in the normal kidney and accumulation in dialysis patients.[8] P-cresol sulfate and indoxyl sulfate have direct vasculotoxic and cytopathic effects and prior studies have suggested an association between the free levels of these solutes and cardiovascular outcomes.[15–25] Hippurate and phenylacetylglutamine also accumulate in dialysis patients but their association with long-term outcomes is not well known. Demonstrating the association of uremic solutes with long-term clinically-relevant outcomes in dialysis patients is an important step in establishing causality between solute accumulation and toxicity and ultimately moving towards reducing toxic levels to improve outcomes.

We measured these four solutes in stored specimens of a national prospective cohort study of incident dialysis patients. The goal of our study was to analyze the longitudinal association between these uremic solutes and cardiovascular morbidity and mortality in dialysis patients.

## Methods

### Study Design

The Choices for Healthy Outcomes in Caring for ESRD (CHOICE) Study is a national prospective cohort of incident dialysis patients.[26] From October 1995 to June 1998, we enrolled 1,041 participants (767 hemodialysis and 274 peritoneal dialysis) from 19 U.S. states, a median of 45 days after initiation of dialysis (95% within 3.5 months). Eligibility criteria were initiation of maintenance dialysis therapy in the preceding 3 months, ability to provide informed consent, age >18 years and ability to speak English or Spanish. We established a specimen bank for all Dialysis Clinic, Inc. (DCI) participants of the CHOICE study. We followed the participants for all-cause mortality through 12/31/2008 and for cardiovascular mortality through 12/31/2004. The Johns Hopkins Medicine Institutional Review Board (Baltimore, Maryland) and the DCI Institutional Review Board approved the study and participants provided written informed consent. Our sub-study sample includes 521 CHOICE participants from 77 dialysis units with available stored specimens.

### Data Collection

#### Specimens

Detailed methods for CHOICE special blood draws have been published previously.[27] Briefly, we collected a non-fasting pre-dialysis blood sample, centrifuged at 2500–3000 revolutions per minute for 15 minutes, separated and refrigerated in the dialysis unit then shipped on ice and stored at −80° Celsius at the DCI central laboratory (Nashville, TN). For this study, we thawed the specimens at the central laboratory, aliquoted and mailed by overnight courier on dry ice to the study laboratory (Stanford University) where we stored them at −80° Celsius until they were thawed for analysis.

#### Uremic Solutes

P-cresol sulfate, indoxyl sulfate, hippurate and phenylacetylglutamine were measured by stable isotope dilution liquid chromatography—tandem mass spectrometry in samples of plasma ultrafiltrate prepared using Nanosep 30K Omega separators (Pall, Ann Arbor, MI), as previously described.[28] The reliability correlation coefficients from masked duplicate specimens were 0.971 for p-cresol sulfate, 0.982 for indoxyl sulfate, 0.977 for hippurate and 0.972 for phenylacetylglutamine.

#### Outcomes

Our primary outcomes were cardiovascular mortality and first cardiovascular event. Secondary outcome was all-cause mortality. We adjudicated mortality using information from clinic report, hospital records, National Death Index, Centers for Medicare & Medicaid Services (CMS) death notification forms, and Social Security records, as previously described.[29] We defined first atherosclerotic cardiovascular event (fatal or nonfatal) as an event due to myocardial infarction, cardiac revascularization procedure, stroke, carotid endarterectomy, extremity gangrene or peripheral revascularization procedure, limb amputation, or abdominal aortic aneurysm repair that occurred after enrolment in the study.[29]

#### Other Covariates

We collected data on participants’ age, sex, race, residual kidney function (self-reported ability to produce >1 cup of urine daily) and body mass index (BMI). We adjudicated baseline comorbidities including prevalent cardiovascular disease by abstraction of dialysis unit records, hospital discharge summaries, medication lists, consultation notes, diagnostic imaging, and cardiac imaging reports and scoring of the Index of Coexistent Disease (ICED) by two trained nurses.

ICED is a validated medical record-derived index that captures both presence and severity of comorbid conditions.[30, 31] It is derived using peak scores from Index of Disease Severity (IDS) and Index of Physical Impairment (IPI). Conditions scored in the IDS include ischemic heart disease, congestive heart failure, arrhythmias, other heart diseases, hypertension, cerebrovascular disease, peripheral vascular disease, diabetes mellitus, respiratory disease, malignancy, hepatobiliary disease, arthritis and gastrointestinal disease. For each patient, IDS is scored as 0 if none, 1 if mild, 2 if symptomatic but controlled with treatment and 3 if moderate with manifestations present despite treatment. Physical impairments scored in the IPI include impairments in circulation, respiration, neurologic function, mental function, urinary elimination, bowel elimination, feeding, ambulation, transfer, vision, hearing and speech. For each patient, IPI is scored as 0 if none, 1 if mild to moderate and 3 if severe. Based on the peak IDS and IPI scores, an ICED level is assigned ranging from 0 to 3 with higher score reflecting greater severity of comorbidities.

We obtained routine laboratory data including serum albumin, creatinine, Kt/V_UREA_ and phosphate from medical records. We assessed residual kidney function in our study in two different ways. First, we used data on self-reported ability to produce >1 cup of urine daily. This information was available for all study participants (N = 394). Second, we had previously measured serum β-trace protein, β2 microglobulin and cystatin C, as markers of residual kidney function, in a different subsample of the CHOICE Study. Of the 394 patients in this study, 206 patients had data available for these low molecular weight proteins. These measurements were performed at the University of Minnesota.

### Statistical Analysis

We compared the baseline characteristics of participants overall and across each solute tertiles using chi-squared tests and linear regression for categorical and continuous variables, respectively. Relationships among solutes and blood urea nitrogen were compared using scatterplots and Pearson and Spearman correlation coefficients.

Our initial review of the data revealed some samples with very high values for free levels of p-cresol sulfate and indoxyl sulfate. Comparison with total p-cresol sulfate and indoxyl sulfate levels measured previously using high pressure liquid chromatography (HPLC)[32] also revealed unusually high free fractions for these solutes. We identified extreme values based on data distribution in an external dataset of 119 freshly collected and processed plasma samples from 43 patients (S1 Table). Because of the possibility that these extreme values might represent problems with sample handling, we excluded samples with extreme values of either p-cresol sulfate or indoxyl sulfate from the primary analysis. However, in sensitivity analyses we tested how robust the finding were when we included the full sample. We defined extreme values as samples with either p-cresol sulfate or indoxyl sulfate above two standard deviation (SD) of the mean based on external data [n = 87 (16.7%)]. Of the remaining samples, we further excluded samples if either percent free p-cresol sulfate or indoxyl sulfate values were >15% of total concentration [n = 40 (7.7%)].

Covariates with missing values included body mass index (5.6%), residual kidney function (3.7%), phosphate (9.4%), creatinine (9.2%), albumin (9.4%) and Kt/V_UREA_ (22.7%). To avoid listwise deletion,[33] we imputed missing data with 10 data replicates using multiple imputation by the chained equations method implemented by the ice program in STATA. We used Cox proportional hazards regression to model the risk of outcomes per SD increase in solute levels, censoring participants at transplantation or end of study period. We adjusted our analyses for demographics (age, sex and race), clinical characteristics (body mass index, residual kidney function as defined by self-reported ability to produce >1 cup of urine daily, ICED score, diabetes and cardiovascular disease) and laboratory tests (albumin, phosphate, creatinine and Kt/V_UREA_). We checked proportional hazards assumptions by tests of Schoenfeld residuals.[34]

In additional analyses, we sought to determine the combined effects of high levels of multiple solutes (combined solute index). We generated this index by first standardizing the value of each solute (mean = 0; SD = 1) followed by categorization of the standardized values into deciles (range 1–10) and then creating an average of the decile category for each participant. We analyzed this combined solute index as a continuous and a categorical (quintiles) variable.

We generated graphical displays of the adjusted hazard of outcomes with the solutes and the combined solute index modeled as restricted cubic spline with 3 knots to allow for the visual assessment of the functional association between solutes and outcomes. We investigated non-linear associations by incorporating splines in our analysis based on these graphical displays. Sensitivity analyses included analyses of the full cohort without excluding extreme values and subgroup analysis of those with available Kt/V_UREA_. In the subgroup of patients (n = 206) with previously measured low molecular weight proteins (serum β-trace protein, β2 microglobulin and cystatin C), we explored further adjustment for residual kidney function by adding these low molecular weight proteins to the primary analysis Cox model. This analysis is therefore adjusted for: demographics (age, sex and race), clinical characteristics (body mass index, residual kidney function as defined by self-reported ability to produce >1 cup of urine daily, ICED score, diabetes and cardiovascular disease), laboratory tests (albumin, phosphate, creatinine and Kt/V_UREA_) and residual kidney function biomarkers (serum β-trace protein, β2 microglobulin and cystatin C).

Statistical analyses were performed using STATA software, version 12.1 (Stata Corp. www.stata.com). Statistical significance was defined as *p*<0.05 using two-tailed tests.

## Results

### Baseline Characteristics

There were 521 hemodialysis participants with available stored samples. After excluding those with extreme values from our primary analysis, our final cohort included 394 participants from 73 dialysis units. Participants included versus excluded were less likely to be white or have cardiovascular disease, had slightly lower urea, hemoglobin and higher albumin (S2 Table). Table 1 describes the overall characteristics of the 394 participants. The average age of the participants was 57 years, 65% were white and 55% were male.

**Table 1:** Baseline Characteristics of 394 Hemodialysis Participants of the CHOICE Study.

Characteristic	Overall
**Solutes Concentration**	
Total P-Cresol Sulfate, mg/dL	
Mean (Standard Deviation)	3.027 (1.493)
Median (25^th^ to 75^th^ Percentiles)	3.008 (1.911–4.068)
Free P-Cresol Sulfate, mg/dL	
Mean (Standard Deviation)	0.196 (0.128)
Median (25^th^ to 75^th^ Percentiles)	0.174 (0.094–0.277)
Total Indoxyl Sulfate, mg/dL	
Mean (Standard Deviation)	1.710 (1.020)
Median (25^th^ to 75^th^ Percentiles)	1.558 (0.992–2.312)
Free Indoxyl Sulfate, mg/dL	
Mean (Standard Deviation)	0.126 (0.100)
Median (25^th^ to 75^th^ Percentiles)	0.097 (0.053–0.172)
Free Hippurate, mg/dL	
Mean (Standard Deviation)	1.5 (2.1)
Median (25^th^ to 75^th^ Percentiles)	0.861 (0.307–1.9)
Free Phenylacetylglutamine, mg/dL	
Mean (Standard Deviation)	2.3 (1.7)
Median (25^th^ to 75^th^ Percentiles)	2.0 (0.998–3.1)
**Demographics**	
Age, years	57.2 (14.9)
White race	255 (64.7)
Male sex	216 (54.8)
**Clinical Characteristics**	
Residual urine output, % with > 1 cup at baseline	316 (83.2)
Body Mass Index, Kg/m^2^	27.6 (6.8)
Attributed Cause of End Stage Renal Disease	
Diabetes mellitus	183 (46.4)
Hypertension	69 (17.5)
Glomerulonephritis	69 (17.5)
Other	73 (18.5)
ICED = 3	105 (26.6)
Diabetes	210 (53.3)
Gastrointestinal Diseases	162 (41.1)
Cardiovascular Disease	204 (51.8)
Congestive Heart Failure	181 (45.9)
Time since start of dialysis, months	5.5 (2.7)
**Laboratory Tests**	
Blood Urea Nitrogen, mg/dL	56.0 (15.6)
Kt/V_UREA_	1.4 (0.286)
Creatinine, mg/dL	7.9 (2.8)
Potassium, mEq/L	4.7 (0.662)
Glucose, mg/dL	164.5 (98.3)
Bicarbonate, mEq/L	20.8 (3.0)
Hemoglobin, g/dL	11.0 (1.3)
Corrected Calcium, mg/dL	9.5 (0.812)
Phosphate, mg/dL	5.4 (1.5)
Albumin, g/dL	3.8 (0.357)
CRP, mg/L (median, 25^th^ – 75^th^ percentiles)	0.392 (0.168–0.947)
IL-6, mg/L (median, 25^th^ – 75^th^ percentiles)	4.1 (2.5–7.4)

Note: Numbers presented are mean (standard deviation) or percent unless otherwise specified.

Conversion factors for units: albumin in g/dL to g/L, x 10; calcium in mg/dL to mmol/L, x 0.2495; phosphate in mg/dL to mmol/L, x 0.3229; hemoglobin in g/dL to g/L, x 10; BUN in mg/dL to urea in mmol/L, x 0.357; creatinine in mg/dL to μmol/L, x 88.4; p-cresol sulfate in mg/dL to μmol/L, x 53.1; indoxyl sulfate in mg/dL to μmol/L, x 46.9; hippuric acid in mg/dL to μmol/L, x 55.8; phenylacetylglutamine in mg/dL to μmol/L, x 37.8.

No conversion is necessary for potassium and bicarbonate in mEq/L to mmol/L.

Abbreviations: ICED: Index of Coexistent Disease Score; Kt/V: dialysis dose (K-dialyzer clearance of urea, t-dialysis time, V-volume of distribution of urea); CRP: C-Reactive Protein; IL-6: Interleukin 6

### Uremic Solutes

**Fig 1:**
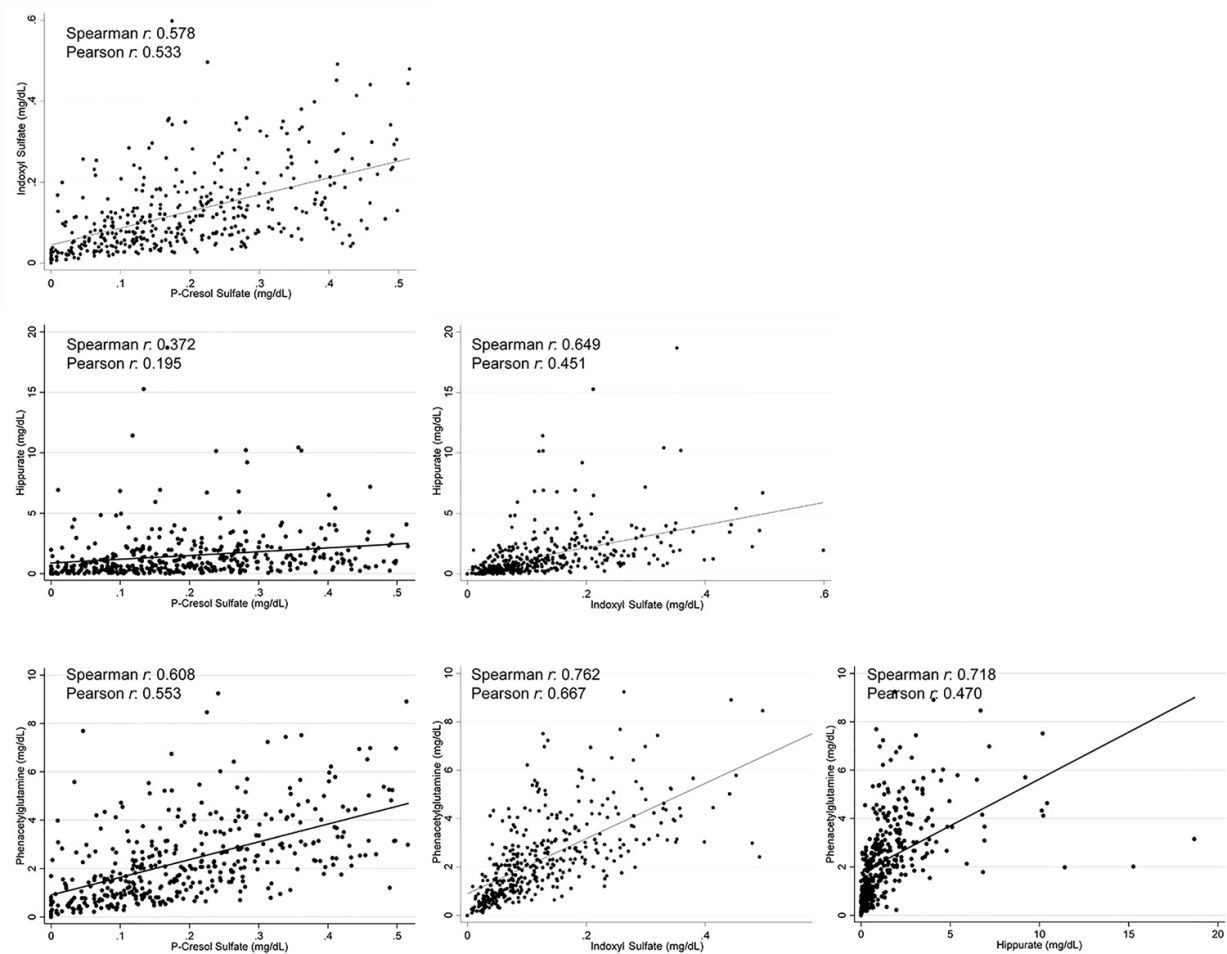
Correlations between Solutes in 394 Incident Hemodialysis Patients of the CHOICE Study. Scatterplots demonstrate the association between the solutes. Dots represent to concentrations of the two solutes on scatterplot. Line represents the linear fit between the two solutes. Spearman and Pearson correlation coefficients are also reported in a text box. P-values for all correlations were ≤0.001 for both Spearman and Pearson correlations.

S3 Table presents the concentrations of solutes overall and after exclusion of extreme values. After exclusion, the free concentrations of all four solutes were lower and had less variance. The correlation between the total and free concentrations of solutes improved after removal of extreme observations. The correlation between total and free p-cresol sulfate improved from 0.525 to 0.832 and between total and free indoxyl sulfate improved from 0.646 to 0.838. There was moderate correlation between all solutes except for p-cresol and hippurate which showed weak correlation (Fig 1). There was weak correlation between the solutes and predialysis urea (S1 Fig). S4 Table describe the characteristics of the 394 participants by tertiles of the solutes. Older age was associated with higher p-cresol sulfate, indoxyl sulfate and phenylacetylglutamine. Those with diabetes and higher random blood glucose were more likely to have lower indoxyl sulfate and hippurate. Higher potassium was associated with higher concentration of all solutes. Higher creatinine, phosphate and albumin were associated with higher concentrations of indoxyl sulfate, hippurate and phenylacetylglutamine. Presence of residual kidney function (self-reported urine output > 1 cup daily) was associated with lower levels of phenylacetylglutamine but none of the other solutes.

### Cardiovascular Mortality

**Fig 2:**
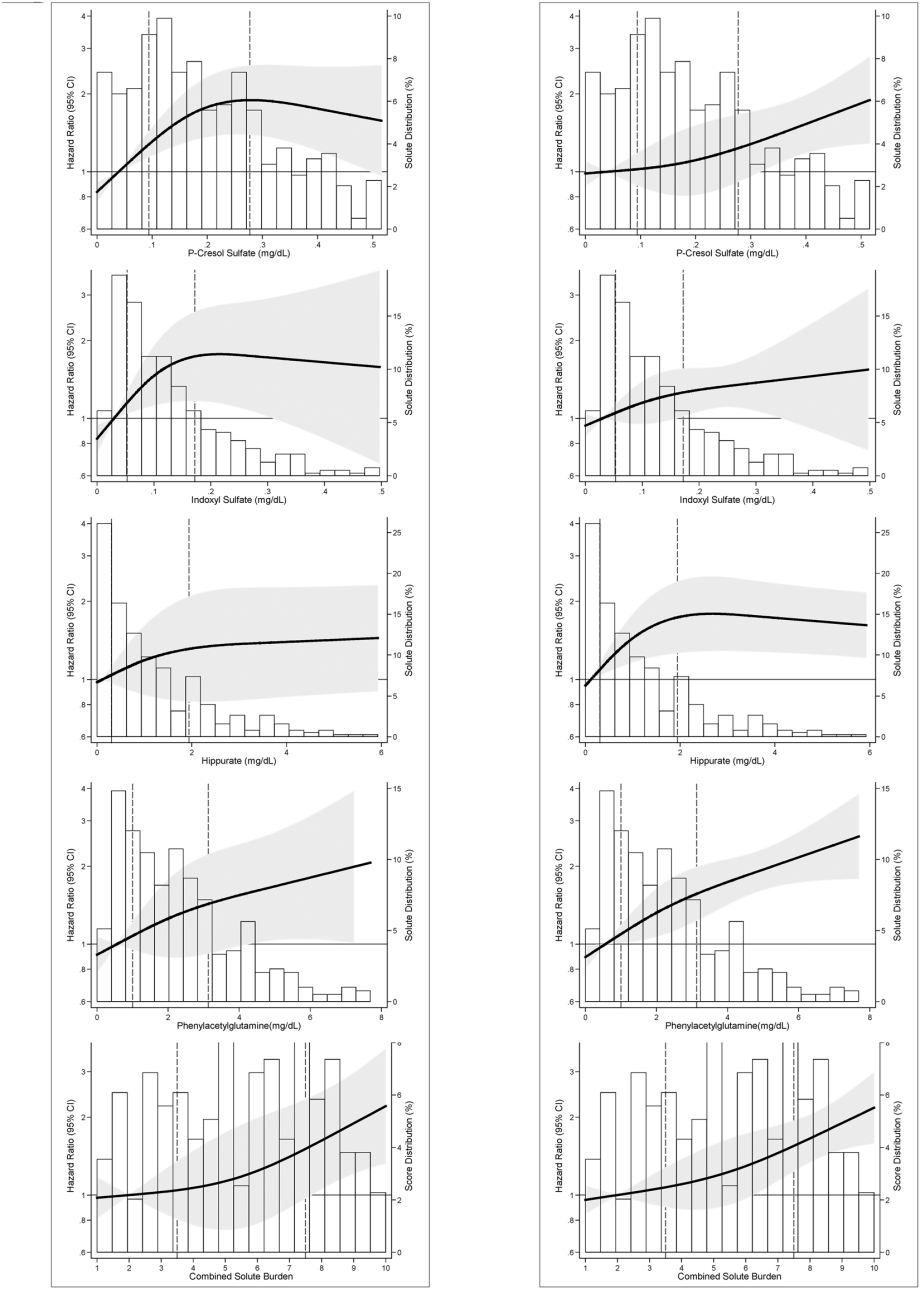
Adjusted Relative Hazard of Outcomes in 394 Incident Hemodialysis Patients of the CHOICE Study. **Panel 2A:** Adjusted hazard of Cardiovascular Mortality. **Panel 2B:** Adjusted hazard of First Cardiovascular Event. Relative hazard predicted using Cox proportional hazards regression adjusted for demographics (age, sex and race), clinical characteristics [body mass index, residual kidney function (self-reported ability to produce >1 cup of urine daily), Index of Coexistent Disease (ICED) score, diabetes and cardiovascular disease] and laboratory tests (Kt/V_UREA_, albumin, phosphate and creatinine). Solutes and combined solute index are modeled as restricted cubic splines with knots at the 10th, 50th, and 90th percentiles. The solid line is the adjusted HR; 10th percentile is used as the reference (HR = 1). The shaded area is the 95% CI of the HR. Bars are the frequency histogram, showing the distribution of each solute and combined solute index. Vertical broken lines mark the 25^th^ and 75^th^ percentile of the distribution.

There were 121 cardiovascular deaths over 1,393 person-years of follow-up (median 3.4 years). In fully adjusted analyses, p-cresol sulfate was associated with a higher risk of cardiovascular death [hazard ratio per SD increase (HR_PerSD_), 1.62; 95% CI, 1.17–2.25; p = 0.004; Table 2]. Fig 2A displays the adjusted hazard of cardiovascular mortality with the solutes. Hippurate and phenylacetylglutamine had a linear increase in risk. P-cresol sulfate had a linear increase in risk below the 75^th^ percentile (HR_PerSD_, 2.57; 95% CI, 1.19–4.75; p = 0.003) with attenuation of the hazard above this level although the change in slope did not reach statistical significance (p-value for change in slope = 0.13; S5 Table). Indoxyl sulfate also had a functional association similar to p-cresol sulfate (HR_PerSD_ below 75^th^ percentile, 1.83; 95% CI, 0.98–3.42; p = 0.06) but the change in slope was not statistically significant (p = 0.08).

**Table 2:** Association of Uremic Solutes with Outcomes among 394 Hemodialysis Participants of the CHOICE Study.

	Model 1 (Unadjusted) ^1^		Model 2 (Minimally Adjusted) ^2^		Model 3 (Fully Adjusted) ^3^	
	HR (95% CI)	p	HR (95% CI)	p	HR (95% CI)	p
**Cardiovascular Mortality**						
P-Cresol Sulfate	**1.85 (1.09–3.16)**	**0.02**	1.51 (0.99–2.29)	0.06	**1.62 (1.17–2.25)**	**0.004**
Indoxyl Sulfate	1.28 (0.91–1.81)	0.16	1.05 (0.69–1.60)	0.82	1.18 (0.79–1.76)	0.43
Hippurate	1.11 (0.98–1.25)	0.10	1.01 (0.91–1.13)	0.83	1.04 (0.92–1.18)	0.51
Phenylacetylglutamine	1.42 (1.07–1.89)	0.02	1.20 (0.90–1.58)	0.21	1.30 (0.96–1.76)	0.10
**First Cardiovascular Event**						
P-Cresol Sulfate	**1.91 (1.38–2.65)**	**<0.001**	**1.63 (1.20–2.23)**	**0.002**	**1.60 (1.23–2.08)**	**<0.001**
Indoxyl Sulfate	1.16 (0.97–1.39)	0.10	1.02 (0.80–1.31)	0.85	1.10 (0.83–1.46)	0.51
Hippurate	1.01 (0.92–1.11)	0.82	0.96 (0.87–1.06)	0.39	1.00 (0.90–1.11)	0.96
Phenylacetylglutamine	**1.43 (1.24–1.64)**	**<0.001**	**1.29 (1.15–1.45)**	**<0.001**	**1.37 (1.18–1.58)**	**<0.001**

*Abbreviations*: HR, Hazard Ratio; CI, Confidence Interval.

Hazard ratio per 1 standard deviation increase in the solute level modeled using Cox proportional hazards regression.

^1^ Model 1: Crude model without adjustment.

^2^ Model 2: Minimally adjusted: HR adjusted for demographics (age, sex and race).

^3^ Model 3: Fully adjusted: HR adjusted for demographics (age, sex and race), clinical characteristics [body mass index, residual kidney function (self-reported ability to produce >1 cup of urine daily), Index of Coexistent Disease (ICED) score, diabetes and cardiovascular disease] and laboratory tests (Kt/V_UREA_, albumin, phosphate and creatinine).

Note: Mean (Standard Deviation) for the free solutes are: P-cresol sulfate 0.196 (0.128) mg/dL; Indoxyl Sulfate 0.126 (0.100) mg/dL; Hippurate 1.5 (2.1) mg/dL and Phenylacetylglutamine 2.3 (1.7) mg/dL.

### First Cardiovascular Event

There were 217 cardiovascular events. In the unadjusted and fully adjusted models, both p-cresol sulfate and phenylacetylglutamine were associated with the risk of first cardiovascular event (Table 2). Fig 2B demonstrates the linear association between hippurate and first cardiovascular event below the 75^th^ percentile (HR_PerSD_, 1.69; 95% CI, 1.11–2.56; p = 0.01) with attenuation of the hazard above this level (p-value for change in slope 0.01; S5 Table).

### Combined Solute Index

The combined solute index was associated with older age and higher urea, creatinine, potassium and phosphate (S6 Table). Those in the highest quintile for combined solute index had a 96% higher risk of cardiovascular death (HR, 1.96; 95% CI, 1.05–3.68; p = 0.04) and a 62% higher risk of first cardiovascular event (HR, 1.62; 95% CI, 1.12–2.35; p = 0.01) compared with those in the lowest quintiles (Table 3 and Fig 2). A combined solute index that includes only p-cresol sulfate and phenylacetylglutamine showed results that were generally similar to the associations with the combined solute index with all four solutes (S7 Table).

**Table 3:** Association of Combined Solute Index Quintiles and Outcomes among 394 Hemodialysis Participants of the CHOICE Study.

		Model 1 (Unadjusted) ^1^		Model 2 (Minimally Adjusted) ^2^		Model 3 (Fully Adjusted) ^3^	
	Range	HR (95% CI)	p	HR (95% CI)		HR (95% CI)	p
**Cardiovascular Mortality**							
Continuous	(1–10)	**1.30 (1.04–1.61)**	**0.02**	1.13 (0.92–1.39)	0.23	**1.20 (1.01–1.43)**	**0.04**
Categorical							
Q1 (Lowest)	(1–3)	Reference		Reference		Reference	
Q2	(3.25–5)	1.47 (0.91–2.36)	0.11	1.20 (0.73–1.96)	0.47	1.34 (0.79–2.26)	0.28
Q3	(5.25–6.25)	**1.79 (1.06–3.02)**	**0.03**	1.65 (0.94–2.92)	0.08	**1.77 (1.02–3.08)**	**0.04**
Q4	(6.5–8)	1.57 (0.99–2.50)	0.06	1.16 (0.77–1.76)	0.47	1.19 (0.70–2.03)	0.52
Q5 (Highest)	(8.25–10)	**2.41 (1.22–4.76)**	**0.01**	1.50 (0.77–2.90)	0.23	**1.96 (1.05–3.68)**	**0.04**
*p-trend*		**0.03**		0.33		0.10	
**First Cardiovascular Event**							
Continuous	(1–10)	**1.19 (1.08–1.32)**	**<0.001**	**1.10 (1.01–1.21)**	**0.04**	**1.19 (1.09–1.31)**	**<0.001**
Categorical							
Q1 (Lowest)	(1–3)	Reference		Reference		Reference	
Q2	(3.25–5)	1.09 (0.78–1.54)	0.61	1.00 (0.71–1.41)	0.99	1.06 (0.75–1.48)	0.75
Q3	(5.25–6.25)	**1.49 (1.11–2.01)**	**0.008**	**1.45 (1.10–1.91)**	**0.009**	**1.70 (1.17–2.48)**	**0.005**
Q4	(6.5–8)	**1.42 (1.05–1.92)**	**0.02**	**1.20 (0.87–1.65)**	**0.27**	**1.45 (1.06–1.98)**	**0.02**
Q5 (Highest)	(8.25–10)	**1.68 (1.16–2.42)**	**0.006**	**1.27 (0.88–1.85)**	**0.20**	**1.62 (1.12–2.35)**	**0.01**
*p-trend*		**<0.001**		**0.04**		**0.001**	

*Abbreviations*: HR, Hazard Ratio; CI, Confidence Interval.

Hazard ratio per 1 standard deviation increase in the combined solute index in the continuous analysis modeled using Cox proportional hazards regression.

Note: Mean (Standard Deviation) for combined solute index is 5.5 (2.4).

^1^ Model 1: Crude model without adjustment.

^2^ Model 2: Minimally adjusted: HR adjusted for demographics (age, sex and race).

^3^ Model 3: Fully adjusted: HR adjusted for demographics (age, sex and race), clinical characteristics [body mass index, residual kidney function (self-reported ability to produce >1 cup of urine daily), Index of Coexistent Disease (ICED) score, diabetes and cardiovascular disease] and laboratory tests (Kt/V_UREA_, albumin, phosphate and creatinine).

NOTE: Combined solute index is calculated as follows

^1)^ Generate standardized value of each solute with a mean of 0 and standard deviation of 1.

^2)^ For each standardized solute create deciles based on percentiles of the data (range 1–10)

^3)^ Calculate the combined solute index by averaging the decile category for each participant

^4)^ Generate quintiles of the combined solute index. The lowest quintile is the reference.

### Secondary Outcome: All-Cause Mortality

There were 236 deaths during follow-up. In fully adjusted models, none of the solutes were associated with a statistically significant association with death although p-cresol, hippurate and phenylacetylglutamine had a trend towards increased risk of death (S8 Table and S2 Fig).

### Sensitivity Analyses

Analysis of the full cohort without excluding extreme observations (n = 521) showed results similar to the primary analysis in terms of magnitude and direction of effect (S9 Table). Restricting primary analysis to the subgroup with available Kt/V_UREA_ (n = 303) also showed similar results (data not shown). In the subset of 206 patients with data on low molecular weight proteins (β-trace protein, β2 microglobulin and cystatin C), measured as markers of residual kidney function, there was weak correlation with p-cresol sulfate (S3 Fig) and moderate correlation with indoxyl sulfate (S4 Fig), hippurate (S5 Fig) and phenylacetylglutamine (S6 Fig). Further adjusting for residual kidney function biomarkers (serum β-trace protein, β2 microglobulin and cystatin C) in addition to the variables in Model 4 [demographics (age, sex and race), clinical characteristics (body mass index, residual kidney function as defined by self-reported ability to produce >1 cup of urine daily, ICED score, diabetes and cardiovascular disease), laboratory tests (albumin, phosphate, creatinine and Kt/V_UREA_)], did not change the direction of effect for p-cresol sulfate and phenylacetylglutamine (S10 Table).

## Discussion

In this report from a US national prospective cohort of incident hemodialysis patients from 73 dialysis centers, we found that colon-derived organic solutes that are removed by the native kidney by secretion had a graded association with cardiovascular morbidity and mortality. Plasma p-cresol sulfate was associated with a 62% higher risk of cardiovascular mortality and a 60% higher risk of first cardiovascular event and plasma phenylacetylglutamine was associated with a 37% higher risk of first cardiovascular event, per 1 SD higher solute concentration. Patients in the highest quintile of combined solute index that incorporated the four solutes measured in this study (p-cresol sulfate, indoxyl sulfate, hippurate and phenylacetylglutamine) had a 96% higher risk of cardiovascular mortality and 62% higher risk of first cardiovascular event compared to those in the lowest quintile. Our findings suggest that these four accumulated non-urea solutes in dialysis patients may be associated with toxicity and, in particular, a higher risk of cardiovascular events.

Advancing kidney failure leads to accumulation of numerous substances that are cleared by the native kidney by glomerular filtration, tubular secretion and tubular uptake. The four solutes studied share the property of colon microbial derivation as well as rapid clearance by secretion in the normal kidney. P-cresol sulfate (187 Da) is the sulfate of p-cresol made through the action of the unusual microbial enzyme 4-hydroxyphenylacetate decarboxylase on 4-hydroxyphenylacetate which is derived from tyrosine and from plant polyphenols.[10–12, 14] Indoxyl sulfate (212 Da) is the sulfate of indoxyl made from indole produced by the action of microbial tryptophanase on tryptophan. Hippurate (178 Da) is the glycine conjugate of benzoic acid which is partially derived from colon microbial action on various plant compounds.[35–38] Phenylacetylglutamine (263 Da) is the glutamine conjugate of phenylacetic acid which is produced almost exclusively by the action of colon microbes on phenylalanine.[13, 39] Conventionally “adequate” dialysis dose guided by Kt/V_UREA_ maintains predialysis urea (60 Da) at a low level but is much less effective in controlling the levels of these other solutes. We recently found that expressed as multiple of their average concentrations in normal subjects, the pre-dialysis free concentrations of p-cresol sulfate (41-fold higher), indoxyl sulfate (111-fold higher), hippurate (108-fold higher) and phenylacetylglutamine (122-fold higher) were strikingly greater than those of urea (5-fold higher) or creatinine (13-fold higher) in hemodialysis patients.[28] P-cresol sulfate accumulation can cause shedding of endothelial microparticles that impair nitric oxide signaling and induce endothelial dysfunction.[22, 23] Indoxyl sulfate accumulation can cause impairment of cellular oxidative systems leading to free radical generation which can cause toxicity in the renal tubular cells and vascular endothelium.[17, 24] There has been less consideration of toxicity from hippurate and phenylacetylglutamine. The current study demonstrates an association between solute accumulation and cardiovascular events suggesting the retained solutes might indeed be toxic.

A number of earlier, smaller, studies have reported an association between free p-cresol sulfate, indoxyl sulfate and outcomes in dialysis patients. In a single center study, free p-cresol sulfate and indoxyl sulfate measured in a cohort of chronic kidney disease patients (including 44 on dialysis) were associated with risk of death.[16, 18] In another single center study of hemodialysis patients (n = 100), free p-cresol sulfate but not indoxyl sulfate was associated with the higher odds of cardiovascular events (odds ratio 1.78; p = 0.01).[21] In a study of 175 hemodialysis patients from two dialysis units, free p-cresol sulfate was associated with risk of death when analyzed as a categorical variable (adjusted HR 2.28 above versus below median; p = 0.02).[15] Another group of investigators reported an association between p-cresol sulfate and risk of cardiovascular events in 50 hemodialysis patients (HR for free p-cresol sulfate, 1.66; p<0.01) and 46 peritoneal dialysis patients (HR for total p-cresol, 1.05; p<1.01).(19, 20) In a single center study of 112 prevalent hemodialysis patients, both free p-cresol sulfate and indoxyl sulfate were associated with the risk of death (HR 1.01 for p-cresol and 1.10 for indoxyl sulfate).[25] We had previously reported in the same cohort as this study that total (free and bound) p-cresol sulfate and indoxyl sulfate were not associated with outcomes in incident dialysis patients.[32] A reanalysis of the total solutes data, restricting to the 394 participants included in this study demonstrated that total p-cresol sulfate was associated with 21% higher risk of cardiovascular mortality per SD higher level (S11 Table). The reasons for the discrepancy between the relation of total and free solutes levels to outcomes cannot be known with certainty. We presume the free level is a better indicator of the potential toxicity of uremic solutes, as tissues are exposed to free solute levels. Confounding by albumin concentration may be another explanation. A higher albumin concentration tends to increase the total solute level for any given free solute level, and higher albumin concentrations are also associated with better outcomes in dialysis patients.[40] It is possible that unmeasured solutes also compete for the same binding sites on albumin. A high total burden of such solutes will tend to impair the binding of individual solutes. As a result, among patients with the same total levels of a particular solute, those with the higher aggregate solute burden would have higher free levels of that solute and measurement only of total solute levels could tend to conceal an association of that solute with poor outcomes.

We noted approximately 25% reduction in the hazard estimates (log HR) for p-cresol sulfate and phenylacetylglutamine compared with no change with hippurate after adjustment for age, sex and race (Table 2, Model 2). Although the direction of effects did not change and the confidence intervals overlap, these findings appear intriguing. They could reflect partial adjustment for comorbidities with adjustment for age as older age is associated with more comorbidities. Older age could also mean a longer cumulative exposure to uremic toxins, perhaps even prior to start of dialysis, leading to higher uremic vasculopathy. Biological differences in sex and race or associated environmental and social determinants may also contribute to variations in solutes. These findings are hypothesis generating and should be confirmed in other studies.

There are some limitations to our study. First, we measured solutes at a single time point and levels of these solutes likely vary over time. Repeated solute measurements might reveal a stronger association of solute levels with outcomes. Second, a particular consideration is that residual native kidney function, which was present in many of our subjects when baseline samples were obtained, can have a large effect on the levels of secreted solutes.[41] In our sensitivity analyses, adjusting for the low molecular weight solutes that reflect residual kidney function did not change the observed association between p-cresol sulfate and outcomes. Third, sample handling is an issue in large scale, representative, effectiveness studies conducted in routine practice. However, our results were robust regardless of excluding outliers. Finally, although our prospective study demonstrates a longitudinal and dose response relation between solutes and outcomes, it does not establish causality. The associations we note in our study could also be reflective of other unknown uremic toxins that are retained along with the four solutes that we measured. Further studies are needed to identify unknown uremic solutes in dialysis patients and determine their mechanism of toxicity. These limitations of our study are balanced by several strengths including a prospective national multicenter design, inclusion of only incident dialysis patients, detailed and precise information on comorbidities and systematic adjudication of outcomes. These comprehensive data allowed us to extensively adjust for potential confounders and reduce the chances of residual confounding. Importantly, our multicenter study cohort provides generalizability of our results to US hemodialysis patients.

In conclusion, in this national prospective study of incident dialysis patients in the US, we demonstrate an association between free levels of colon-derived organic solutes that are normally removed by the native kidney by secretion and cardiovascular outcomes. Our findings suggest that these solutes that are not adequately cleared by conventional dialysis may contribute to morbidity and mortality in dialysis patients. Replication of our findings in other large prospective cohorts will provide further scientific evidence to pursue clinical trials targeted to reducing solute production, improving dialytic removal or both.[3, 4, 6, 42–52]

## Supporting Information

S1 Fig Correlations between Predialysis Urea and Solutes in 303 Incident Hemodialysis Patients of the CHOICE Study. Scatterplots demonstrate the association between predialysis urea and solutes. Dots represent to concentrations of the two solutes on scatterplot. Line represents the linear fit between the two solutes. Spearman and Pearson correlation coefficients are also reported in a text box. P-values for all correlations were ≤0.001 for both Spearman and Pearson correlations.
(TIFF)

S2 Fig Adjusted Relative Hazard of All-Cause Mortality in 394 Incident Hemodialysis Patients of the CHOICE Study. Relative hazard predicted using Cox proportional hazards regression adjusted for demographics (age, sex and race), clinical characteristics [body mass index, residual kidney function (self-reported ability to produce >1 cup of urine daily), Index of Coexistent Disease (ICED) score, diabetes and cardiovascular disease] and laboratory tests (Kt/V_UREA_, albumin, phosphate and creatinine). Solutes and combined solute index are modeled as restricted cubic splines with knots at the 10th, 50th, and 90th percentiles. The solid line is the adjusted HR; 10th percentile is used as the reference (HR = 1). The shaded area is the 95% CI of the HR. Bars are the frequency histogram, showing the distribution of each solute and combined solute index. Vertical broken lines mark the 25^th^ and 75^th^ percentile of the distribution.
(TIFF)

S3 Fig Correlations between P-Cresol Sulfate and Low Molecular Weight Proteins in 206 Incident Hemodialysis Patients of the CHOICE Study. Scatterplots demonstrate the association of p-cresol sulfate with β-Trace Protein, β2 microglobulin and cystatin C. Dots represent concentrations of the two solutes on scatterplot. Line represents the linear fit between the two solutes. Spearman and Pearson correlation coefficients are also reported in a text box.
(TIFF)

S4 Fig Correlations between Indoxyl Sulfate and Low Molecular Weight Proteins in 206 Incident Hemodialysis Patients of the CHOICE Study. Scatterplots demonstrate the association of indoxyl sulfate with β-Trace Protein, β2 microglobulin and cystatin C. Dots represent concentrations of the two solutes on scatterplot. Line represents the linear fit between the two solutes. Spearman and Pearson correlation coefficients are also reported in a text box.
(TIFF)

S5 Fig Correlations between Indoxyl Sulfate and Low Molecular Weight Proteins in 206 Incident Hemodialysis Patients of the CHOICE Study. Scatterplots demonstrate the association of indoxyl sulfate with β-Trace Protein, β2 microglobulin and cystatin C. Dots represent concentrations of the two solutes on scatterplot. Line represents the linear fit between the two solutes. Spearman and Pearson correlation coefficients are also reported in a text box.
(TIFF)

S6 Fig Correlations between Hippurate and Low Molecular Weight Proteins in 206 Incident Hemodialysis Patients of the CHOICE Study. Scatterplots demonstrate the association of hippurate with β-Trace Protein, β2 microglobulin and cystatin C. Dots represent concentrations of the two solutes on scatterplot. Line represents the linear fit between the two solutes. Spearman and Pearson correlation coefficients are also reported in a text box.
(TIFF)

S1 Table P-Cresol Sulfate and Indoxyl Sulfate Levels from 43 Patients with Freshly Collected and Processed Specimens. (DOCX)

S2 Table Comparison of the Baseline Characteristics of the Included versus Excluded Participants. (DOCX)

S3 Table Concentrations of Solutes Before and After Excluding Extreme Observations. (DOCX)

S4 Table Baseline Characteristics of 394 Hemodialysis Participants of the CHOICE Study by Tertiles of P-Cresol Sulfate (S4A_Table), Indoxyl Sulfate (S4B_Table), Hippurate (S4C_Table) and Phenylacetylglutamine (S4D_Table). (DOCX)

S5 Table Spline Models and Outcomes among 394 Hemodialysis Participants of the CHOICE Study. (DOCX)

S6 Table Baseline Characteristics of 394 Hemodialysis Participants of the CHOICE Study by Quintiles of Solute Score. (DOCX)

S7 Table Association of Combined Solute Index and Outcomes among 394 Hemodialysis Participants of the CHOICE Study (Model 3, Fully Adjusted). (DOCX)

S8 Table Association of Uremic Solutes with All-Cause Mortality among 394 Hemodialysis Participants of the CHOICE Study. (DOCX)

S9 Table Association of Uremic Solutes and Outcomes among 521 Hemodialysis Participants of the CHOICE Study (Without Excluding Extreme Values). (DOCX)

S10 Table Association of Uremic Solutes with Outcomes among 206 Hemodialysis Participants of the CHOICE Study (With Data on Low Molecular Weight Proteins). (DOCX)

S11 Table Comparison of Total Solutes Results with Previous Analysis of Total Solute Levels and Outcomes. (DOCX)
