# Supplementary material for: Free Levels of Selected Organic Solutes and Cardiovascular Morbidity and Mortality in Hemodialysis Patients: Results from the Retained Organic Solutes and Clinical Outcomes (ROSCO) Investigators
Source: PLoS One. 2015 May 4;10(5):e0126048. doi: 10.1371/journal.pone.0126048 (PMC4418712; doi:10.1371/journal.pone.0126048)
Supplement: S1 Table — (DOCX) [file pone.0126048.s007.docx]

**S1 Table: P-Cresol Sulfate and Indoxyl Sulfate Levels from 43 Patients with Freshly Collected and Processed Specimens**

|  | **P-Cresol Sulfate, mg/dL** | | | **Indoxyl Sulfate, mg/dL** | | |
| --- | --- | --- | --- | --- | --- | --- |
|  | **Total** | **Free** | **% Free** | **Total** | **Free** | **% Free** |
| **Number of Samples** | 119 | 119 | 119 | 119 | 119 | 119 |
| **Range** | 0.128, 6.928 | 0.003, 0.646 | 2.019, 27.319 | 0.91, 5.627 | 0.048, 1.07 | 3.45, 26.909 |
| **Mean (Standard Deviation)** | 3.215 (1.409) | 0.24 (0.141) | 7.193 (2.78) | 3.181 (1.139) | 0.289 (0.175) | 8.891 (3.454) |
| **Median**  **(25th to 75th Percentile)** | 3.326  (2.203, 4.16) | 0.22  (0.152, 0.309) | 6.978  (5.4, 8.737) | 3.1  (2.428, 3.929) | 0.253  (0.166, 0.365) | 7.945  (6.339, 11.114) |

Conversion factors for units: p-cresol sulfate in mg/dL to μmol/L, x 53.1; indoxyl sulfate in mg/dL to μmol/L, x 46.9; hippuric acid in mg/dL to μmol/L, x 55.8; phenylacetylglutamine in mg/dL to μmol/L, x 37.8.
