# Supplementary material for: Free Levels of Selected Organic Solutes and Cardiovascular Morbidity and Mortality in Hemodialysis Patients: Results from the Retained Organic Solutes and Clinical Outcomes (ROSCO) Investigators
Source: PLoS One. 2015 May 4;10(5):e0126048. doi: 10.1371/journal.pone.0126048 (PMC4418712; doi:10.1371/journal.pone.0126048)
Supplement: S2 Table — (DOCX) [file pone.0126048.s008.docx]

**S2 Table: Comparison of the Baseline Characteristics of the Included versus Excluded Participants***

| **Characteristic** | **Overall** | **Available Samples** | **p^1^** | **After Excluding Extreme Values** | **p^2^** | **p^3^** |
| --- | --- | --- | --- | --- | --- | --- |
| **Numbers** | 1041 | 521 |  | 394 |  |  |
| **Demographics** |  |  |  |  |  |  |
| Age, years | 57.9 (15.0) | 58.3 (14.7) | 0.51 | 57.2 (14.9) | 0.26 | 0.02 |
| White | 746 (71.7) | 348 (66.8) | <0.001 | 255 (64.7) | <0.001 | 0.08 |
| Male | 564 (54.2) | 282 (54.1) | 0.97 | 216 (54.8) | 0.75 | 0.58 |
|  |  |  |  |  |  |  |
| **Clinical Characteristics** |  |  |  |  |  |  |
| Body Mass Index, Kg/m^2^ | 27.1 (6.7) | 27.4 (7.0) | 0.12 | 27.6 (6.8) | 0.06 | 0.48 |
| Cause of End Stage Renal Disease |  |  | 0.41 |  | 0.76 | 0.33 |
| Diabetes mellitus | 481 (46.5) | 249 (47.8) |  | 183 (46.4) |  |  |
| Hypertension | 180 (17.4) | 95 (18.2) |  | 69 (17.5) |  |  |
| Glomerulonephritis | 168 (16.2) | 84 (16.1) |  | 69 (17.5) |  |  |
| Other | 205 (19.8) | 93 (17.9) |  | 73 (18.5) |  |  |
| ICED=3 | 308 (29.6) | 152 (29.2) | 0.09 | 105 (26.6) | 0.11 | 0.11 |
| Diabetes | 561 (54.0) | 286 (54.9) | 0.56 | 210 (53.3) | 0.73 | 0.20 |
| Cardiovascular Disease | 593 (57.1) | 293 (56.2) | 0.59 | 204 (51.8) | 0.007 | <0.001 |
| Congestive Heart Failure | 486 (46.8) | 257 (49.3) | 0.10 | 181 (45.9) | 0.67 | 0.006 |
|  |  |  |  |  |  |  |
| **Laboratory Tests** |  |  |  |  |  |  |
| Blood Urea Nitrogen, mg/dL | 56.7 (16.3) | 55.3 (14.4) | 0.001 | 54.9 (14.8) | 0.007 | 0.35 |
| Kt/V_UREA_ | 1.4 (0.426) | 1.3 (0.302) | 0.14 | 1.3 (0.292) | 0.08 | 0.17 |
| Creatinine, mg/dL | 7.3 (2.5) | 7.3 (2.4) | 0.71 | 7.3 (2.4) | 0.43 | 0.69 |
| Potassium, mEq/L | 4.5 (0.589) | 4.5 (0.538) | <0.001 | 4.5 (0.532) | 0.04 | 0.08 |
| Glucose, mg/dL | 167.7 (87.4) | 168.7 (86.8) | 0.67 | 165.6 (82.0) | 0.49 | 0.23 |
| Hemoglobin, g/dL | 10.8 (1.3) | 10.5 (1.2) | <0.001 | 10.6 (1.2) | 0.001 | 0.21 |
| Corrected Calcium, mg/dL | 9.4 (0.657) | 9.4 (0.621) | 0.86 | 9.4 (0.637) | 0.69 | 0.12 |
| Phosphate, mg/dL | 5.2 (1.3) | 5.3 (1.2) | 0.30 | 5.2 (1.2) | 0.76 | 0.45 |
| Albumin, g/dL | 3.6 (0.374) | 3.6 (0.354) | 0.07 | 3.7 (0.344) | 0.01 | 0.07 |

Note: Numbers presented are mean (standard deviation) or percent unless otherwise specified.

Conversion factors for units: albumin in g/dL to g/L, x 10; calcium in mg/dL to mmol/L, x 0.2495; phosphate in mg/dL to mmol/L, x 0.3229; hemoglobin in g/dL to g/L, x 10; BUN in mg/dL to urea in mmol/L, x 0.357; creatinine in mg/dL to umol/L, x 88.4; No conversion is necessary for potassium in mEq/L to mmol/L.

P-values are by Student t-test for continuous variables and chi-square test for categorical variables

Abbreviations: Kt/V_UREA_: dialysis dose (K-dialyzer clearance of urea, t-dialysis time, V-volume of distribution of urea)

* Laboratory data in this table are baseline values from the first 3 months after enrolment in CHOICE Study and may differ from Table 1.

^1^ P-value for comparison between overall cohort and those with available samples (N=521).

^2^ P-value for comparison between overall cohort and those without extreme values (N=394).

^3^ P-value for comparison between available samples (521) and those without extreme values (N=394).
