# Supplementary material for: Free Levels of Selected Organic Solutes and Cardiovascular Morbidity and Mortality in Hemodialysis Patients: Results from the Retained Organic Solutes and Clinical Outcomes (ROSCO) Investigators
Source: PLoS One. 2015 May 4;10(5):e0126048. doi: 10.1371/journal.pone.0126048 (PMC4418712; doi:10.1371/journal.pone.0126048)
Supplement: S3 Table — (DOCX) [file pone.0126048.s009.docx]

**S3 Table: Concentrations of Solutes Before and After Excluding Extreme Observations**

|  |  | **All Patients** | **Included** | **Excluded** | ***p*** |
| --- | --- | --- | --- | --- | --- |
| **Solutes** |  | 521 | 394 | 127 |  |
| **P-Cresol Sulfate, mg/dL** | **Total** | 3.18 (1.76) | 3.03 (1.49) | 3.65 (2.35) | 0.005 |
|  | **Free** | 0.31 (0.36) | 0.20 (0.13) | 0.66 (0.57) | <0.001 |
| **Indoxyl Sulfate, mg/dL** | **Total** | 1.81 (1.11) | 1.71 (1.02) | 2.13 (1.32) | 0.001 |
|  | **Free** | 0.19 (0.20) | 0.13 (0.10) | 0.39 (0.29) | <0.001 |
| **Hippurate, mg/dL** | **Free** | 1.69 (2.15) | 1.49 (2.07) | 2.30 (2.28) | 0.001 |
| **Phenylacetylglutamine, mg/dL** | **Free** | 2.97 (2.71) | 2.34 (1.70) | 4.93 (4.03) | <0.001 |

Conversion factors for units: p-cresol sulfate in mg/dL to μmol/L, x 53.1; indoxyl sulfate in mg/dL to μmol/L, x 46.9; hippuric acid in mg/dL to μmol/L, x 55.8; phenylacetylglutamine in mg/dL to μmol/L, x 37.8.

Note: Concentrations are presented as mean (standard deviation). P-values are by Student two-sample t test with unequal variances.
