# Supplementary material for: Free Levels of Selected Organic Solutes and Cardiovascular Morbidity and Mortality in Hemodialysis Patients: Results from the Retained Organic Solutes and Clinical Outcomes (ROSCO) Investigators
Source: PLoS One. 2015 May 4;10(5):e0126048. doi: 10.1371/journal.pone.0126048 (PMC4418712; doi:10.1371/journal.pone.0126048)
Supplement: S4 Table — (DOCX) [file pone.0126048.s010.docx]

**Table A: Baseline Characteristics of 394 Hemodialysis Participants of the CHOICE Study by Tertiles of P-Cresol Sulfate**

| **Characteristic** | **Lowest** | **Middle** | **Highest** | **p-trend** |
| --- | --- | --- | --- | --- |
| **Numbers** | 132 (33.5) | 131 (33.2) | 131 (33.2) |  |
| **Free P-Cresol Sulfate, mg/dL** |  |  |  |  |
| Range | 0.000-0.120 | 0.121-0.245 | 0.245-0.516 |  |
| Mean (Standard Deviation) | 0.063 (0.037) | 0.177 (0.037) | 0.347 (0.076) |  |
|  |  |  |  |  |
| **Demographics** |  |  |  |  |
| Age, years | 53.6 (14.5) | 57.3 (14.8) | 60.6 (14.9) | <0.001 |
| White | 82 (62.1) | 87 (66.4) | 86 (65.6) | 0.74 |
| Male | 74 (56.1) | 67 (51.1) | 75 (57.3) | 0.57 |
|  |  |  |  |  |
| **Clinical Characteristics** |  |  |  |  |
| Residual urine output, > 1 cup at baseline | 106 (82.2) | 111 (88.1) | 99 (79.2) | 0.16 |
| Body Mass Index, Kg/m^2^ | 27.7 (7.2) | 28.2 (7.1) | 26.7 (6.2) | 0.18 |
| Cause of End Stage Renal Disease |  |  |  | 0.41 |
| Diabetes mellitus | 60 (45.5) | 59 (45.0) | 64 (48.9) |  |
| Hypertension | 20 (15.2) | 21 (16.0) | 28 (21.4) |  |
| Glomerulonephritis | 28 (21.2) | 26 (19.8) | 15 (11.5) |  |
| Other | 24 (18.2) | 25 (19.1) | 24 (18.3) |  |
| ICED=3 | 37 (28.0) | 32 (24.4) | 36 (27.5) | 0.57 |
| Diabetes | 67 (50.8) | 68 (51.9) | 75 (57.3) | 0.53 |
| Gastrointestinal Diseases | 56 (42.4) | 54 (41.2) | 52 (39.7) | 0.92 |
| Cardiovascular Disease | 61 (46.2) | 71 (54.2) | 72 (55.0) | 0.29 |
| Congestive Heart Failure | 53 (40.2) | 61 (46.6) | 67 (51.1) | 0.20 |
| Time since start of dialysis, months | 5.4 (2.5) | 5.4 (2.8) | 5.7 (2.8) | 0.32 |
|  |  |  |  |  |
| **Laboratory Tests** |  |  |  |  |
| Blood Urea Nitrogen, mg/dL | 52.2 (15.2) | 54.6 (17.3) | 60.6 (13.1) | 0.002 |
| Kt/V_UREA_ | 1.3 (0.290) | 1.4 (0.272) | 1.4 (0.293) | 0.12 |
| Creatinine, mg/dL | 7.8 (3.4) | 7.8 (2.4) | 8.3 (2.7) | 0.18 |
| Potassium, mEq/L | 4.6 (0.653) | 4.7 (0.637) | 4.8 (0.679) | 0.004 |
| Glucose, mg/dL | 153.2 (86.8) | 176.1 (122.1) | 165.3 (83.3) | 0.39 |
| Bicarbonate, mEq/L | 20.6 (3.4) | 21.0 (2.8) | 20.9 (2.7) | 0.45 |
| Hemoglobin, g/dL | 11.0 (1.4) | 11.0 (1.3) | 11.1 (1.3) | 0.44 |
| Corrected Calcium, mg/dL | 9.5 (0.841) | 9.5 (0.905) | 9.5 (0.692) | 0.66 |
| Phosphate, mg/dL | 5.3 (1.5) | 5.7 (1.7) | 5.3 (1.4) | 0.80 |
| Albumin, g/dL | 3.7 (0.391) | 3.7 (0.344) | 3.8 (0.336) | 0.82 |
| CRP, mg/L (median, 25^th^ – 75^th^ percentiles) | 0.422 (0.176-1.0) | 0.390 (0.154-0.972) | 0.365 (0.168-0.725) | 0.58 |
| IL-6, mg/L (median, 25^th^ – 75^th^ percentiles) | 4.0 (2.3-7.4) | 4.1 (2.6-7.3) | 4.4 (2.6-7.4) | 0.94 |

Note: Numbers presented are mean (standard deviation) or percent unless otherwise specified.

Conversion factors for units: albumin in g/dL to g/L, x 10; calcium in mg/dL to mmol/L, x 0.2495; phosphate in mg/dL to mmol/L, x 0.3229; hemoglobin in g/dL to g/L, x 10; BUN in mg/dL to urea in mmol/L, x 0.357; creatinine in mg/dL to umol/L, x 88.4; p-cresol sulfate in mg/dL to μmol/L, x 53.1. No conversion is necessary for potassium and bicarbonate in mEq/L to mmol/L.

Abbreviations: ICED: Index of Coexistent Disease Score; Kt/V_UREA_: dialysis dose (K-dialyzer clearance of urea, t-dialysis time, V-volume of distribution of urea); CRP: C-Reactive Protein; IL-6: Interleukin 6

**Table B: Baseline Characteristics of 394 Hemodialysis Participants of the CHOICE Study by Tertiles of Indoxyl Sulfate**

| **Characteristic** | **Lowest** | **Middle** | **Highest** | **p-trend** |
| --- | --- | --- | --- | --- |
| **Numbers** | 132 (33.5) | 131 (33.2) | 131 (33.2) |  |
| **Free Indoxyl Sulfate, mg/dL** |  |  |  |  |
| Range | 0.000-0.068 | 0.069-0.138 | 0.139-0.599 |  |
| Mean (Standard Deviation) | 0.040 (0.016) | 0.100 (0.021) | 0.240 (0.089) |  |
|  |  |  |  |  |
| **Demographics** |  |  |  |  |
| Age, years | 55.5 (14.5) | 56.9 (14.6) | 59.1 (15.6) | 0.05 |
| White | 79 (59.8) | 81 (61.8) | 95 (72.5) | 0.07 |
| Male | 76 (57.6) | 69 (52.7) | 71 (54.2) | 0.72 |
|  |  |  |  |  |
| **Clinical Characteristics** |  |  |  |  |
| Residual urine output, > 1 cup at baseline | 111 (87.4) | 107 (84.3) | 98 (77.8) | 0.11 |
| Body Mass Index, Kg/m^2^ | 27.8 (7.4) | 27.8 (6.7) | 27.0 (6.4) | 0.35 |
| Cause of End Stage Renal Disease |  |  |  | 0.003 |
| Diabetes mellitus | 65 (49.2) | 76 (58.0) | 42 (32.1) |  |
| Hypertension | 24 (18.2) | 18 (13.7) | 27 (20.6) |  |
| Glomerulonephritis | 22 (16.7) | 16 (12.2) | 31 (23.7) |  |
| Other | 21 (15.9) | 21 (16.0) | 31 (23.7) |  |
| ICED=3 | 34 (25.8) | 41 (31.3) | 30 (22.9) | 0.41 |
| Diabetes | 70 (53.0) | 80 (61.1) | 60 (45.8) | 0.05 |
| Gastrointestinal Diseases | 54 (40.9) | 57 (43.5) | 51 (38.9) | 0.96 |
| Cardiovascular Disease | 68 (51.5) | 68 (51.9) | 68 (51.9) | 1.00 |
| Congestive Heart Failure | 61 (46.2) | 64 (48.9) | 56 (42.7) | 0.61 |
| Time since start of dialysis, months | 5.3 (2.4) | 5.4 (2.4) | 5.9 (3.2) | 0.16 |
|  |  |  |  |  |
| **Laboratory Tests** |  |  |  |  |
| Blood Urea Nitrogen, mg/dL | 52.1 (16.9) | 56.1 (15.6) | 59.5 (13.5) | <0.001 |
| Kt/V_UREA_ | 1.4 (0.255) | 1.3 (0.304) | 1.4 (0.297) | 1.00 |
| Creatinine, mg/dL | 6.6 (2.4) | 8.1 (2.8) | 9.2 (2.8) | <0.001 |
| Potassium, mEq/L | 4.5 (0.561) | 4.8 (0.623) | 4.8 (0.737) | 0.004 |
| Glucose, mg/dL | 163.7 (94.6) | 182.8 (122.9) | 146.5 (65.7) | 0.02 |
| Bicarbonate, mEq/L | 21.2 (3.2) | 20.4 (3.0) | 20.9 (2.7) | 0.72 |
| Hemoglobin, g/dL | 11.0 (1.4) | 11.0 (1.4) | 11.1 (1.2) | 0.54 |
| Corrected Calcium, mg/dL | 9.5 (0.760) | 9.5 (0.742) | 9.6 (0.932) | 0.61 |
| Phosphate, mg/dL | 5.1 (1.4) | 5.5 (1.4) | 5.7 (1.7) | 0.03 |
| Albumin, g/dL | 3.7 (0.388) | 3.7 (0.330) | 3.8 (0.336) | 0.002 |
| CRP, mg/L (median, 25^th^ – 75^th^ percentiles) | 0.428 (0.175-0.976) | 0.365 (0.186-0.952) | 0.385 (0.146-0.788) | 0.79 |
| IL-6, mg/L (median, 25^th^ – 75^th^ percentiles) | 3.9 (2.4-6.8) | 4.4 (2.6-7.8) | 4.1 (2.5-6.9) | 0.38 |

Note: Numbers presented are mean (standard deviation) or percent unless otherwise specified.

Conversion factors for units: albumin in g/dL to g/L, x 10; calcium in mg/dL to mmol/L, x 0.2495; phosphate in mg/dL to mmol/L, x 0.3229; hemoglobin in g/dL to g/L, x 10; BUN in mg/dL to urea in mmol/L, x 0.357; creatinine in mg/dL to umol/L, x 88.4; indoxyl sulfate in mg/dL to μmol/L, x 46.9. No conversion is necessary for potassium and bicarbonate in mEq/L to mmol/L.

Abbreviations: ICED: Index of Coexistent Disease Score; Kt/V_UREA_: dialysis dose (K-dialyzer clearance of urea, t-dialysis time, V-volume of distribution of urea); CRP: C-Reactive Protein; IL-6: Interleukin 6

**Table C: Baseline Characteristics of 394 Hemodialysis Participants of the CHOICE Study by Tertiles of Hippurate**

| **Characteristic** | **Lowest** | **Middle** | **Highest** | **p-trend** |
| --- | --- | --- | --- | --- |
| **Numbers** | 132 (33.5) | 131 (33.2) | 131 (33.2) |  |
| **Free Hippurate, mg/dL** |  |  |  |  |
| Range | 0.000-0.479 | 0.490-1.4 | 1.4-18.7 |  |
| Mean (Standard Deviation) | 0.214 (0.136) | 0.891 (0.271) | 3.4 (2.7) |  |
|  |  |  |  |  |
| **Demographics** |  |  |  |  |
| Age, years | 56.7 (13.5) | 57.2 (15.4) | 57.6 (16.0) | 0.61 |
| White | 77 (58.3) | 82 (62.6) | 96 (73.3) | 0.03 |
| Male | 66 (50.0) | 77 (58.8) | 73 (55.7) | 0.35 |
|  |  |  |  |  |
| **Clinical Characteristics** |  |  |  |  |
| Residual urine output, > 1 cup at baseline | 113 (86.9) | 98 (79.7) | 105 (82.7) | 0.30 |
| Body Mass Index, Kg/m^2^ | 28.5 (7.7) | 27.5 (6.8) | 26.6 (5.9) | 0.11 |
| Cause of End Stage Renal Disease |  |  |  | 0.10 |
| Diabetes mellitus | 72 (54.5) | 64 (48.9) | 47 (35.9) |  |
| Hypertension | 20 (15.2) | 20 (15.3) | 29 (22.1) |  |
| Glomerulonephritis | 18 (13.6) | 25 (19.1) | 26 (22.1) |  |
| Other | 22 (16.7) | 22 (16.8) | 29 (22.1) |  |
| ICED=3 | 33 (25.0) | 37 (28.2) | 35 (26.7) | 0.30 |
| Diabetes | 80 (60.6) | 71 (54.2) | 59 (45.0) | 0.04 |
| Gastrointestinal Diseases | 56 (42.4) | 53 (40.5) | 53 (40.5) | 0.19 |
| Cardiovascular Disease | 71 (53.8) | 68 (51.9) | 65 (49.6) | 0.80 |
| Congestive Heart Failure | 64 (48.5) | 55 (42.0) | 62 (47.3) | 0.53 |
| Time since start of dialysis, months | 5.1 (1.9) | 5.3 (2.4) | 6.1 (3.4) | 0.04 |
|  |  |  |  |  |
| **Laboratory Tests** |  |  |  |  |
| Blood Urea Nitrogen, mg/dL | 53.1 (18.4) | 54.8 (13.9) | 59.8 (13.5) | <0.001 |
| Kt/V_UREA_ | 1.4 (0.306) | 1.4 (0.286) | 1.3 (0.267) | 0.38 |
| Creatinine, mg/dL | 6.3 (1.9) | 8.2 (3.0) | 9.3 (2.7) | <0.001 |
| Potassium, mEq/L | 4.4 (0.551) | 4.6 (0.682) | 5.0 (0.650) | <0.001 |
| Glucose, mg/dL | 177.4 (103.9) | 170.2 (119.2) | 147.0 (65.0) | 0.008 |
| Bicarbonate, mEq/L | 21.3 (3.3) | 20.5 (2.8) | 20.6 (2.7) | 0.28 |
| Hemoglobin, g/dL | 10.9 (1.5) | 11.1 (1.2) | 11.0 (1.3) | 0.75 |
| Corrected Calcium, mg/dL | 9.5 (0.626) | 9.5 (0.863) | 9.6 (0.922) | 0.46 |
| Phosphate, mg/dL | 4.9 (1.3) | 5.4 (1.3) | 5.9 (1.8) | <0.001 |
| Albumin, g/dL | 3.7 (0.371) | 3.8 (0.325) | 3.8 (0.361) | 0.01 |
| CRP, mg/L (median, 25^th^ – 75^th^ percentiles) | 0.471 (0.184-1.1) | 0.381 (0.173-0.809) | 0.349 (0.161-0.854) | 0.75 |
| IL-6, mg/L (median, 25^th^ – 75^th^ percentiles) | 4.1 (2.6-7.3) | 4.1 (2.7-7.1) | 4.2 (2.4-7.4) | 0.28 |

Note: Numbers presented are mean (standard deviation) or percent unless otherwise specified.

Conversion factors for units: albumin in g/dL to g/L, x 10; calcium in mg/dL to mmol/L, x 0.2495; phosphate in mg/dL to mmol/L, x 0.3229; hemoglobin in g/dL to g/L, x 10; BUN in mg/dL to urea in mmol/L, x 0.357; creatinine in mg/dL to umol/L, x 88.4; hippuric acid in mg/dL to μmol/L, x 55.8. No conversion is necessary for potassium and bicarbonate in mEq/L to mmol/L.

Abbreviations: ICED: Index of Coexistent Disease Score; Kt/V_UREA_: dialysis dose (K-dialyzer clearance of urea, t-dialysis time, V-volume of distribution of urea); CRP: C-Reactive Protein; IL-6: Interleukin 6

**Table D: Baseline Characteristics of 394 Hemodialysis Participants of the CHOICE Study by Tertiles of Phenylacetylglutamine**

| **Characteristic** | **Lowest** | **Middle** | **Highest** | **p-trend** |
| --- | --- | --- | --- | --- |
| **Numbers** | 132 (33.5) | 131 (33.2) | 131 (33.2) |  |
| **Free Phenylacetylglutamine, mg/dL** |  |  |  |  |
| Range | 0.000-1.3 | 1.3-2.7 | 2.7-9.2 |  |
| Mean (Standard Deviation) | 0.743 (0.311) | 2.0 (0.406) | 4.3 (1.4) |  |
|  |  |  |  |  |
| **Demographics** |  |  |  |  |
| Age, years | 55.9 (14.2) | 55.2 (15.5) | 60.5 (14.6) | 0.009 |
| White | 79 (59.8) | 92 (70.2) | 84 (64.1) | 0.21 |
| Male | 76 (57.6) | 69 (52.7) | 71 (54.2) | 0.72 |
|  |  |  |  |  |
| **Clinical Characteristics** |  |  |  |  |
| Residual urine output, > 1 cup at baseline | 113 (87.6) | 112 (88.9) | 91 (72.8) | 0.001 |
| Body Mass Index, Kg/m^2^ | 28.0 (7.3) | 27.7 (6.9) | 26.9 (6.3) | 0.37 |
| Cause of End Stage Renal Disease |  |  |  | 0.56 |
| Diabetes mellitus | 65 (49.2) | 65 (49.6) | 53 (40.5) |  |
| Hypertension | 20 (15.2) | 21 (16.0) | 28 (21.4) |  |
| Glomerulonephritis | 24 (18.2) | 24 (18.3) | 21 (16.0) |  |
| Other | 23 (17.4) | 21 (16.0) | 29 (22.1) |  |
| ICED=3 | 32 (24.2) | 40 (30.5) | 33 (25.2) | 0.10 |
| Diabetes | 71 (53.8) | 75 (57.3) | 64 (48.9) | 0.39 |
| Gastrointestinal Diseases | 61 (46.2) | 47 (35.9) | 54 (41.2) | 0.32 |
| Cardiovascular Disease | 69 (52.3) | 67 (51.1) | 68 (51.9) | 0.98 |
| Congestive Heart Failure | 57 (43.2) | 61 (46.6) | 63 (48.1) | 0.72 |
| Time since start of dialysis, months | 5.2 (2.3) | 5.4 (2.4) | 6.0 (3.3) | 0.03 |
|  |  |  |  |  |
| **Laboratory Tests** |  |  |  |  |
| Blood Urea Nitrogen, mg/dL | 50.8 (16.1) | 57.3 (15.5) | 59.5 (14.0) | <0.001 |
| Kt/V_UREA_ | 1.3 (0.286) | 1.4 (0.277) | 1.4 (0.296) | 0.73 |
| Creatinine, mg/dL | 6.6 (2.3) | 7.9 (2.6) | 9.3 (2.9) | <0.001 |
| Potassium, mEq/L | 4.4 (0.573) | 4.8 (0.603) | 4.9 (0.713) | <0.001 |
| Glucose, mg/dL | 168.7 (99.2) | 170.5 (116.7) | 154.8 (76.1) | 0.09 |
| Bicarbonate, mEq/L | 20.9 (3.2) | 21.1 (2.9) | 20.4 (2.7) | 0.16 |
| Hemoglobin, g/dL | 10.9 (1.5) | 11.2 (1.3) | 11.1 (1.2) | 0.35 |
| Corrected Calcium, mg/dL | 9.5 (0.776) | 9.5 (0.739) | 9.6 (0.913) | 0.47 |
| Phosphate, mg/dL | 5.0 (1.4) | 5.7 (1.4) | 5.6 (1.7) | 0.02 |
| Albumin, g/dL | 3.7 (0.346) | 3.7 (0.364) | 3.8 (0.360) | 0.26 |
| CRP, mg/L (median, 25^th^ – 75^th^ percentiles) | 0.418 (0.175-1.0) | 0.396 (0.167-0.947) | 0.356 (0.168-0.725) | 0.84 |
| IL-6, mg/L (median, 25^th^ – 75^th^ percentiles) | 3.8 (2.5-6.7) | 4.1 (2.4-7.4) | 4.5 (2.6-7.4) | 0.28 |

Note: Numbers presented are mean (standard deviation) or percent unless otherwise specified.

Conversion factors for units: albumin in g/dL to g/L, x 10; calcium in mg/dL to mmol/L, x 0.2495; phosphate in mg/dL to mmol/L, x 0.3229; hemoglobin in g/dL to g/L, x 10; BUN in mg/dL to urea in mmol/L, x 0.357; creatinine in mg/dL to umol/L, x 88.4; phenylacetylglutamine in mg/dL to μmol/L, x 37.8.

No conversion is necessary for potassium and bicarbonate in mEq/L to mmol/L.

Abbreviations: ICED: Index of Coexistent Disease Score; Kt/V_UREA_: dialysis dose (K-dialyzer clearance of urea, t-dialysis time, V-volume of distribution of urea); CRP: C-Reactive Protein; IL-6: Interleukin 6
