# Supplementary material for: Free Levels of Selected Organic Solutes and Cardiovascular Morbidity and Mortality in Hemodialysis Patients: Results from the Retained Organic Solutes and Clinical Outcomes (ROSCO) Investigators
Source: PLoS One. 2015 May 4;10(5):e0126048. doi: 10.1371/journal.pone.0126048 (PMC4418712; doi:10.1371/journal.pone.0126048)
Supplement: S5 Table — (DOCX) [file pone.0126048.s011.docx]

**S5 Table: Spline Models and Outcomes among 394 Hemodialysis Participants of the CHOICE Study**

|  | **HR (95% CI)** | **p** | **p for change in slope** |
| --- | --- | --- | --- |
| **Cardiovascular Mortality** |  |  |  |
| P-cresol sulfate |  |  |  |
| Below 75^th^ percentile | 2.57 (1.39-4.75) | 0.003 | 0.13 |
| Above 75^th^ percentile | 0.75 (0.24-2.36) | 0.62 |  |
| Indoxyl Sulfate |  |  |  |
| Below 75^th^ percentile | 1.83 (0.98-3.42) | 0.06 | 0.08 |
| Above 75^th^ percentile | 0.86 (0.49-1.51) | 0.60 |  |
| Hippurate |  |  |  |
| Below 75^th^ percentile | 1.21 (0.58-2.51) | 0.62 | 0.67 |
| Above 75^th^ percentile | 1.02 (0.90-1.15) | 0.78 |  |
| Phenylacetylglutamine |  |  |  |
| Below 75^th^ percentile | 1.36 (0.80-2.32) | 0.26 | 0.82 |
| Above 75^th^ percentile | 1.26 (0.85-1.86) | 0.26 |  |
| Combined solute index |  |  |  |
| Below Median | 1.16 (0.73-1.87) | 0.53 | 0.17 |
| Above Median | 5.04 (0.78-32.35) | 0.09 |  |
| **First Cardiovascular Event** |  |  |  |
| P-cresol sulfate |  |  |  |
| Below 75^th^ percentile | 1.35 (0.81-2.26) | 0.25 | 0.48 |
| Above 75^th^ percentile | 2.12 (0.90-4.99) | 0.08 |  |
| Indoxyl Sulfate |  |  |  |
| Below 75^th^ percentile | 1.30 (0.86-1.98) | 0.21 | 0.53 |
| Above 75^th^ percentile | 0.98 (0.55-1.74) | 0.94 |  |
| Hippurate |  |  |  |
| Below 75^th^ percentile | 1.69 (1.11-2.56) | 0.01 | 0.01 |
| Above 75^th^ percentile | 0.91 (0.81-1.03) | 0.14 |  |
| Phenylacetylglutamine |  |  |  |
| Below 75^th^ percentile | 1.42 (1.03-1.94) | 0.03 | 0.85 |
| Above 75^th^ percentile | 1.33 (0.94-1.89) | 0.11 |  |
| Combined solute index |  |  |  |
| Below Median | 1.35 (1.03-1.79) | 0.27 | 0.47 |
| Above Median | 2.47 (0.58-10.62) | 0.22 |  |

*Abbreviations:* HR, Hazard Ratio; CI, Confidence Interval.

Hazard ratio per 1 standard deviation (overall) increase in the solute level modeled using Cox proportional hazards regression.

HR adjusted for demographics (age, sex and race), clinical characteristics [body mass index, residual kidney function (self-reported ability to produce >1 cup of urine daily), Index of Coexistent Disease (ICED) score, diabetes and cardiovascular disease] and laboratory tests (Kt/V_UREA_, albumin, phosphate and creatinine).
