# Supplementary material for: Free Levels of Selected Organic Solutes and Cardiovascular Morbidity and Mortality in Hemodialysis Patients: Results from the Retained Organic Solutes and Clinical Outcomes (ROSCO) Investigators
Source: PLoS One. 2015 May 4;10(5):e0126048. doi: 10.1371/journal.pone.0126048 (PMC4418712; doi:10.1371/journal.pone.0126048)
Supplement: S6 Table — (DOCX) [file pone.0126048.s012.docx]

**S6 Table: Baseline Characteristics of 394 Hemodialysis Participants of the CHOICE Study by Quintiles of Solute Score**

| **Characteristic** | **Quintile 1**  **(Lowest )** | **Quintile 2** | **Quintile 3** | **Quintile 4** | **Quintile 5**  **(Highest** | **p-trend** |
| --- | --- | --- | --- | --- | --- | --- |
| **Numbers** | 81 (20.6) | 86 (21.8) | 70 (17.8) | 89 (22.6) | 68 (17.3) |  |
| **Score** |  |  |  |  |  |  |
| Range | 1, 3 | 3.25, 5.0 | 5.25, 6.25 | 6.5, 8.0 | 8.25, 10 |  |
| Mean (Standard Deviation) | 2.1 (0.7) | 4.1 (0.6) | 5.7 (0.4) | 7.2 (0.5) | 8.9 (0.6) |  |
|  |  |  |  |  |  |  |
| **Demographics** |  |  |  |  |  |  |
| Age, years | 53.8 (13.6) | 57.0 (14.6) | 54.8 (15.3) | 59.4 (14.4) | 60.9 (16.3) | <0.001 |
| White | 51 (63.0) | 54 (62.8) | 38 (54.3) | 61 (68.5) | 51 (75.0) | 0.12 |
| Male | 46 (56.8) | 46 (53.5) | 37 (52.9) | 47 (52.8) | 40 (58.8) | 0.93 |
|  |  |  |  |  |  |  |
| **Clinical Characteristics** |  |  |  |  |  |  |
| Residual urine output, > 1 cup at baseline | 70 (88.6) | 70 (84.3) | 58 (86.6) | 70 (81.4) | 48 (73.8) | 0.16 |
| Body Mass Index, Kg/m^2^ | 28.8 (8.1) | 26.9 (5.8) | 28.4 (7.7) | 27.3 (6.5) | 26.3 (5.7) | 0.24 |
| Cause of End Stage Renal Disease |  |  |  |  |  | 0.31 |
| Diabetes mellitus | 38 (46.9) | 46 (53.5) | 38 (54.3) | 37 (41.6) | 24 (35.3) |  |
| Hypertension | 13 (16.0) | 14 (16.3) | 7 (10.0) | 22 (24.7) | 13 (19.1) |  |
| Glomerulonephritis | 15 (18.5) | 14 (16.3) | 11 (15.7) | 12 (13.5) | 17 (25.0) |  |
| Other | 15 (18.5) | 12 (14.0) | 14 (20.0) | 18 (20.2) | 14 (20.6) |  |
| ICED=3 | 20 (24.7) | 24 (27.9) | 23 (32.9) | 25 (28.1) | 13 (19.1) | 0.43 |
| Diabetes | 42 (51.9) | 50 (58.1) | 40 (57.1) | 49 (55.1) | 29 (42.6) | 0.34 |
| Gastrointestinal Diseases | 38 (46.9) | 33 (38.4) | 30 (42.9) | 35 (39.3) | 26 (38.2) | 0.69 |
| Cardiovascular Disease | 43 (53.1) | 40 (46.5) | 37 (52.9) | 53 (60.0) | 31 (45.6) | 0.37 |
| Congestive Heart Failure | 35 (43.2) | 39 (45.3) | 34 (48.6) | 47 (52.8) | 26 (38.2) | 0.44 |
| Time since start of dialysis, months | 5.1 (2.0) | 5.3 (2.4) | 5.5 (2.7) | 5.3 (2.4) | 6.5 (3.8) | 0.05 |
|  |  |  |  |  |  |  |
| **Laboratory Tests** |  |  |  |  |  |  |
| Blood Urea Nitrogen, mg/dL | 50.8 (17.6) | 52.3 (14.3) | 57.5 (17.9) | 58.6 (13.2) | 60.9 (13.3) | <0.001 |
| Kt/V_UREA_ | 1.3 (0.262) | 1.4 (0.300) | 1.3 (0.327) | 1.4 (0.270) | 1.4 (0.266) | 0.47 |
| Creatinine, mg/dL | 6.3 (2.5) | 7.3 (2.4) | 8.4 (3.0) | 8.6 (2.8) | 9.5 (2.6) | <0.001 |
| Potassium, mEq/L | 4.4 (0.623) | 4.5 (0.535) | 4.8 (0.655) | 4.8 (0.572) | 4.9 (0.804) | <0.001 |
| Glucose, mg/dL | 158.5 (90.7) | 185.6 (135.4) | 169.9 (99.6) | 160.5 (83.8) | 145.3 (56.3) | 0.19 |
| Bicarbonate, mEq/L | 20.9 (3.7) | 21.0 (2.7) | 20.6 (3.1) | 20.8 (2.7) | 20.6 (2.6) | 0.51 |
| Hemoglobin, mg/dL | 10.8 (1.4) | 11.2 (1.4) | 10.9 (1.4) | 11.1 (1.1) | 11.2 (1.3) | 0.17 |
| Corrected Calcium, mg/dL | 9.5 (0.827) | 9.5 (0.762) | 9.5 (0.689) | 9.6 (0.755) | 9.5 (1.0) | 0.71 |
| Phosphate, mg/dL | 4.9 (1.5) | 5.3 (1.3) | 5.7 (1.3) | 5.6 (1.7) | 5.7 (1.7) | 0.02 |
| Albumin, mg/dL | 3.7 (0.384) | 3.7 (0.367) | 3.8 (0.330) | 3.8 (0.341) | 3.8 (0.350) | 0.07 |
| CRP, mg/L (median, 25^th^ – 75^th^ percentiles) | 0.460 (0.229-1.3) | 0.431 (0.167-0.985) | 0.336 (0.137-0.720) | 0.358 (0.161-1.2) | 0.390 (0.216-0.670) | 0.45 |
| IL-6, mg/L (median, 25^th^ – 75^th^ percentiles) | 3.9 (2.6-6.7) | 3.8 (2.4-7.6) | 4.2 (2.8-6.9) | 4.5 (2.6-7.4) | 4.1 (2.4-6.8) | 0.98 |

Note: Numbers presented are mean (standard deviation) or percent unless otherwise specified.

Conversion factors for units: albumin in g/dL to g/L, x 10; calcium in mg/dL to mmol/L, x 0.2495; phosphate in mg/dL to mmol/L, x 0.3229; hemoglobin in g/dL to g/L, x 10; BUN in mg/dL to urea in mmol/L, x 0.357; creatinine in mg/dL to umol/L, x 88.4; No conversion is necessary for potassium and bicarbonate in mEq/L to mmol/L.

Abbreviations: ICED: Index of Coexistent Disease Score; Kt/V_UREA_: dialysis dose (K-dialyzer clearance of urea, t-dialysis time, V-volume of distribution of urea); CRP: C-Reactive Protein; IL-6: Interleukin 6
