# Supplementary material for: Free Levels of Selected Organic Solutes and Cardiovascular Morbidity and Mortality in Hemodialysis Patients: Results from the Retained Organic Solutes and Clinical Outcomes (ROSCO) Investigators
Source: PLoS One. 2015 May 4;10(5):e0126048. doi: 10.1371/journal.pone.0126048 (PMC4418712; doi:10.1371/journal.pone.0126048)
Supplement: S7 Table — (DOCX) [file pone.0126048.s013.docx]

**S7 Table: Association of Combined Solute Index and Outcomes among 394 Hemodialysis Participants of the CHOICE Study (Model 3, Fully Adjusted)**

|  | **Index calculated from all 4 Solutes** | | **Index using only p-cresol sulfate and phenylacetylglutamine** | |
| --- | --- | --- | --- | --- |
|  | **HR (95% CI)** | **p** | **HR (95% CI)** | **p** |
| **All-Cause Mortality** | | | | |
| Continuous | 1.12 (0.94-1.33) | 0.21 | 1.14 (1.01-1.29) | 0.04 |
| Categorical |  |  |  |  |
| Q1 (Lowest) | Reference |  | Reference |  |
| Q2 | 1.14 (0.79-1.65) | 0.49 | 1.69 (1.06-2.70) | 0.03 |
| Q3 | 1.78 (1.27-2.48) | 0.001 | 1.24 (0.86-1.79) | 0.26 |
| Q4 | 1.23 (0.77-1.95) | 0.38 | 1.47 (0.90-2.40) | 0.12 |
| Q5 (Highest) | 1.31 (0.77-2.24) | 0.32 | 1.64 (1.08-2.50) | 0.02 |
| *p-trend* | 0.36 | | 0.06 | |
| **Cardiovascular Mortality** | | | | |
| Continuous | 1.20 (1.01-1.43) | 0.04 | 1.23 (1.09-1.41) | 0.001 |
| Categorical |  |  |  |  |
| Q1 (Lowest) | Reference |  | Reference |  |
| Q2 | 1.34 (0.79-2.26) | 0.28 | 1.35 (0.75-2.42) | 0.31 |
| Q3 | 1.77 (1.02-3.08) | 0.04 | 1.06 (0.67-1.67) | 0.80 |
| Q4 | 1.19 (0.70-2.03) | 0.52 | 1.20 (0.65-2.21) | 0.55 |
| Q5 (Highest) | 1.96 (1.05-3.68) | 0.04 | 2.01 (1.29-3.14) |  |
| *p-trend* | 0.10 | | 0.001 |  |
| **First Cardiovascular Event** | | | | |
| Continuous | 1.19 (1.09-1.31) | <0.001 | 1.21 (1.12-1.31) | <0.001 |
| Categorical |  |  |  |  |
| Q1 (Lowest) | Reference |  | Reference |  |
| Q2 | 1.06 (0.75-1.48) | 0.75 | 0.86 (0.57-1.30) | 0.47 |
| Q3 | 1.70 (1.17-2.48) | 0.005 | 1.19 (0.74-1.91) | 0.48 |
| Q4 | 1.45 (1.06-1.98) | 0.02 | 1.46 (1.06-2.01) | 0.02 |
| Q5 (Highest) | 1.62 (1.12-2.35) | 0.01 | 1.58 (1.18-2.12) | 0.002 |
| *p-trend* | 0.001 | | <0.001 | |

*Abbreviations:* HR, Hazard Ratio; CI, Confidence Interval.

Hazard ratio per 1 standard deviation increase in the combined solute index modeled using Cox proportional hazards regression adjusted for demographics (age, sex and race), clinical characteristics [body mass index, residual kidney function (self-reported ability to produce >1 cup of urine daily), Index of Coexistent Disease (ICED) score, diabetes and cardiovascular disease] and laboratory tests (Kt/V_UREA_, albumin, phosphate and creatinine).
