# Supplementary material for: Free Levels of Selected Organic Solutes and Cardiovascular Morbidity and Mortality in Hemodialysis Patients: Results from the Retained Organic Solutes and Clinical Outcomes (ROSCO) Investigators
Source: PLoS One. 2015 May 4;10(5):e0126048. doi: 10.1371/journal.pone.0126048 (PMC4418712; doi:10.1371/journal.pone.0126048)
Supplement: S8 Table — (DOCX) [file pone.0126048.s014.docx]

**S8 Table: Association of Uremic Solutes with All-Cause Mortality among 394 Hemodialysis Participants of the CHOICE Study**

|  | **Model 1**  **(Unadjusted) ^1^** | | **Model 2**  **(Minimally Adjusted) ^2^** | | **Model 3**  **(Fully Adjusted) ^3^** | |
| --- | --- | --- | --- | --- | --- | --- |
|  | **HR (95% CI)** | **p** | **HR (95% CI)** | **p** | **HR (95% CI)** | **p** |
| **P-Cresol Sulfate** | 1.43 (0.97-2.11) | 0.07 | 1.25 (0.92-1.71) | 0.16 | 1.27 (0.95-1.70) | 0.11 |
| **Indoxyl Sulfate** | 1.21 (0.94-1.56) | 0.14 | 1.03 (0.76-1.40) | 0.85 | 1.09 (0.77-1.54) | 0.62 |
| **Hippurate** | 1.11 (1.02-1.20) | 0.01 | 1.02 (0.95-1.10) | 0.53 | 1.06 (0.98-1.15) | 0.15 |
| **Phenylacetylglutamine** | 1.32 (1.05-1.65) | 0.02 | 1.15 (0.96-1.39) | 0.14 | 1.17 (0.97-1.42) | 0.10 |
|  |  |  |  |  |  |  |
| **Combined Solute Index** |  |  |  |  |  |  |
| Q1 (Lowest) | Reference |  | Reference |  | Reference |  |
| Q2 | 1.32 (0.95-1.84) | 0.10 | 1.09 (0.79-1.52) | 0.59 | 1.14 (0.79-1.65) | 0.49 |
| Q3 | 1.80 (1.28-2.54) | 0.001 | 1.73 (1.28-2.34) | <0.001 | 1.78 (1.27-2.48) | 0.001 |
| Q4 | 1.51 (0.98-2.33) | 0.06 | 1.17 (0.78-1.76) | 0.44 | 1.23 (0.77-1.95) | 0.38 |
| Q5 (Highest) | 1.72 (1.02-2.91) | 0.04 | 1.18 (0.70-2.00) | 0.54 | 1.31 (0.77-2.24) | 0.32 |
| *p-trend* |  | 0.05 |  | 0.46 |  | 0.36 |

*Abbreviations:* HR, Hazard Ratio; CI, Confidence Interval.

Hazard ratio per 1 standard deviation increase in the solute level modeled using Cox proportional hazards regression.

^1^ Model 1: Crude model without adjustment.

^2^ Model 2: Minimally adjusted: HR adjusted for demographics (age, sex and race).

^3^ Model 3: Fully adjusted: HR adjusted for demographics (age, sex and race), clinical characteristics [body mass index, residual kidney function (self-reported ability to produce >1 cup of urine daily), Index of Coexistent Disease (ICED) score, diabetes and cardiovascular disease] and laboratory tests (Kt/V_UREA_, albumin, phosphate and creatinine).

NOTE: Combined solute index is calculated as follows

1) Generate standardized value of each solute with a mean of 0 and standard deviation of 1.

2) For each standardized solute create deciles based on percentiles of the data (range 1-10)

3) Calculate the combined solute index by averaging the decile category for each participant

4) Generate quintiles of the combined solute index. The lowest quintile is the reference.
