# Supplementary material for: Free Levels of Selected Organic Solutes and Cardiovascular Morbidity and Mortality in Hemodialysis Patients: Results from the Retained Organic Solutes and Clinical Outcomes (ROSCO) Investigators
Source: PLoS One. 2015 May 4;10(5):e0126048. doi: 10.1371/journal.pone.0126048 (PMC4418712; doi:10.1371/journal.pone.0126048)
Supplement: S9 Table — (DOCX) [file pone.0126048.s015.docx]

**S9 Table: Association of Uremic Solutes and Outcomes among 521 Hemodialysis Participants of the CHOICE Study (Without Excluding Extreme Values)**

|  | **Model 1 (Unadjusted)^1^** | | **Model 2 (Adjusted for Age, Sex and Race)^2^** | | **Model 3 (Fully Adjusted)^3^** | |
| --- | --- | --- | --- | --- | --- | --- |
|  | **HR (95% CI)** | **p** | **HR (95% CI)** | **p** | **HR (95% CI)** | **p** |
| **All-Cause Mortality** |  |  |  |  |  |  |
| P-Cresol Sulfate | 1.25 (1.16-1.34) | <0.001 | 1.14 (1.05-1.24) | 0.002 | 1.06 (0.97-1.16) | 0.19 |
| Indoxyl Sulfate | 1.14 (1.02-1.26) | 0.02 | 1.05 (0.95-1.17) | 0.33 | 1.12 (1.02-1.22) | 0.02 |
| Hippurate | 1.08 (1.00-1.17) | 0.07 | 1.00 (0.92-1.09) | 0.97 | 1.07 (0.98-1.16) | 0.11 |
| Phenylacetylglutamine | 1.22 (1.11-1.34) | <0.001 | 1.14 (1.01-1.27) | 0.03 | 1.18 (1.06-1.31) | 0.002 |
| **Cardiovascular Mortality** |  |  |  |  |  |  |
| P-Cresol Sulfate | 1.27 (1.16-1.40) | <0.001 | 1.16 (1.03-1.31) | 0.02 | 1.06 (0.94-1.19) | 0.36 |
| Indoxyl Sulfate | 1.12 (0.95-1.33) | 0.17 | 1.03 (0.85-1.24) | 0.76 | 1.07 (0.92-1.23) | 0.39 |
| Hippurate | 1.05 (0.92-1.20) | 0.46 | 0.97 (0.84-1.11) | 0.64 | 1.05 (0.91-1.20) | 0.52 |
| Phenylacetylglutamine | 1.19 (1.01-1.40) | 0.04 | 1.09 (0.89-1.33) | 0.40 | 1.13 (0.96-1.32) | 0.15 |
| **First Cardiovascular Event** |  |  |  |  |  |  |
| P-Cresol Sulfate | 1.28 (1.19-1.38) | <0.001 | 1.21 (1.11-1.32) | <0.001 | 1.12 (1.01-1.24) | 0.04 |
| Indoxyl Sulfate | 1.15 (1.02-1.28) | 0.02 | 1.09 (0.97-1.23) | 0.14 | 1.17 (1.07-1.28) | 0.001 |
| Hippurate | 1.01 (0.92-1.11) | 0.80 | 0.96 (0.88-1.04) | 0.31 | 1.03 (0.95-1.12) | 0.47 |
| Phenylacetylglutamine | 1.15 (1.00-1.32) | 0.04 | 1.09 (0.95-1.24) | 0.22 | 1.14 (0.96-1.34) | 0.13 |

*Abbreviations:* HR, Hazard Ratio; CI, Confidence Interval.

Hazard ratio per 1 standard deviation increase in the solute level modeled using Cox proportional hazards regression.

^1^ Model 1: Crude model without adjustment.

^2^ Model 2: HR adjusted for demographics (age, sex and race).

^3^ HR adjusted for demographics (age, sex and race), clinical characteristics [body mass index, residual kidney function (self-reported ability to produce >1 cup of urine daily), Index of Coexistent Disease (ICED) score, diabetes and cardiovascular disease] and laboratory tests (Kt/V_UREA_, albumin, phosphate and creatinine).
