# Supplementary material for: Free Levels of Selected Organic Solutes and Cardiovascular Morbidity and Mortality in Hemodialysis Patients: Results from the Retained Organic Solutes and Clinical Outcomes (ROSCO) Investigators
Source: PLoS One. 2015 May 4;10(5):e0126048. doi: 10.1371/journal.pone.0126048 (PMC4418712; doi:10.1371/journal.pone.0126048)
Supplement: S10 Table — (DOCX) [file pone.0126048.s016.docx]

**S10 Table: Association of Uremic Solutes with Outcomes among 206 Hemodialysis Participants of the CHOICE Study (With Data on Low Molecular Weight Proteins)**

|  | **Model 3**  **(Fully Adjusted) ^1^** | | **Model 4**  **(Model 3 + Adj for BTP, B2M, Cystatin C) ^2^** | |
| --- | --- | --- | --- | --- |
|  | **HR (95% CI)** | **p** | **HR (95% CI)** | **p** |
| **Cardiovascular Mortality** |  |  |  |  |
| P-Cresol Sulfate | 1.41 (0.73-2.72) | 0.30 | 1.36 (0.76-2.46) | 0.30 |
| Indoxyl Sulfate | 0.78 (0.48-1.27) | 0.31 | 0.59 (0.37-0.95) | 0.03 |
| Hippurate | 1.31 (0.98-1.31) | 0.09 | 1.13 (0.95-1.35) | 0.17 |
| Phenylacetylglutamine | 1.41 (1.06-1.87) | 0.02 | 1.33 (0.98-1.81) | 0.07 |
| **First Cardiovascular Event** |  |  |  |  |
| P-Cresol Sulfate | 1.64 (0.98-2.72) | 0.06 | 1.60 (0.93-2.74) | 0.09 |
| Indoxyl Sulfate | 0.80 (0.55-1.16) | 0.24 | 0.72 (0.45-1.14) | 0.16 |
| Hippurate | 0.99 (0.88-1.11) | 0.83 | 0.99 (0.87-1.12) | 0.88 |
| Phenylacetylglutamine | 1.26 (0.96-1.65) | 0.09 | 1.34 (0.92-1.98) | 0.13 |

*Abbreviations:* HR, Hazard Ratio; CI, Confidence Interval.

Hazard ratio per 1 standard deviation increase in the solute level modeled using Cox proportional hazards regression.

^1^ Model 3: Fully adjusted: HR adjusted for demographics (age, sex and race), clinical characteristics [body mass index, residual kidney function (self-reported ability to produce >1 cup of urine daily), Index of Coexistent Disease (ICED) score, diabetes and cardiovascular disease] and laboratory tests (Kt/V_UREA_, albumin, phosphate and creatinine).

^2^ Model 4: Includes all variables in Model 3 + serum β–trace protein (BTP), β2 microglobulin (B2M) and cystatin C.
