# Supplementary material for: Free Levels of Selected Organic Solutes and Cardiovascular Morbidity and Mortality in Hemodialysis Patients: Results from the Retained Organic Solutes and Clinical Outcomes (ROSCO) Investigators
Source: PLoS One. 2015 May 4;10(5):e0126048. doi: 10.1371/journal.pone.0126048 (PMC4418712; doi:10.1371/journal.pone.0126048)
Supplement: S11 Table — (DOCX) [file pone.0126048.s017.docx]

**S11 Table: Comparison of Total Solutes Results with Previous Analysis of Total Solute Levels and Outcomes**

|  |  |  | **All-Cause Mortality** | | **CVD Mortality** | |
| --- | --- | --- | --- | --- | --- | --- |
|  | **N** | **Models** | **P-Cresol Sulfate** | **Indoxyl Sulfate** | **P-Cresol Sulfate** | **Indoxyl Sulfate** |
| **Previous Paper**  **(Melamed et al)** | 521 | 1 | 1.02 (0.90-1.16) | 1.04 (0.91-1.18) | 1.07 (0.88-1.29) | 0.96 (0.79-1.18) |
| **This Paper** | 394 | 2 | 1.03 (0.92-1.15) | 0.97 (0.81-1.16) | 1.19 (0.99-1.43) | 1.02 (0.82-1.26) |
|  | 394 | 3 | 1.03 (0.90-1.17) | 0.96 (0.79-1.15) | 1.21 (1.01-1.46) | 1.00 (0.80-1.24) |

Hazard ratio (95% CI) per 1 standard deviation increase in the solute level modeled using Cox proportional hazards regression.

Model 1: As presented in Melamed et al. Adjusted for age, sex, race, comorbidity score (ICED), baseline albumin, BMI and creatinine.

Model 2: Subset of 394 patients after exclusion of those with extreme values (see methods for details). Adjusted for same variables as Model 1.

Model 3: Subset of 394 patients after exclusion of those with extreme values (see methods for details). Adjusted for demographics (age, sex and race), clinical characteristics [body mass index, residual kidney function (self-reported ability to produce >1 cup of urine daily), Index of Coexistent Disease (ICED) score, diabetes and cardiovascular disease] and laboratory tests (Kt/V_UREA_, albumin, phosphate and creatinine).
